# Critical Role of the Acetylene Content and Fe/C Ratio on the Thickness and Density of Vertically Aligned Carbon Nanotubes Grown at Low Temperature by a One-Step Catalytic Chemical Vapor Deposition Process

**DOI:** 10.3390/nano12142338

**Published:** 2022-07-07

**Authors:** Antoine Combrisson, Emeline Charon, Mathieu Pinault, Cécile Reynaud, Martine Mayne-L’Hermite

**Affiliations:** Nanoscience et Innovation pour les Matériaux, la Biomédecine et l’Energie, Commissariat à l’Energie Atomique, Centre National de la Recherche Scientifique, Université Paris-Saclay, 91191 Gif-sur-Yvette, France; antoine.combrisson@cea.fr (A.C.); mathieu.pinault@cea.fr (M.P.); cecile.reynaud@cea.fr (C.R.); martine.mayne@cea.fr (M.M.-L.)

**Keywords:** carbon nanotubes, synthesis, aerosol-assisted CVD, aluminum substrate, growth rate, VACNT thickness, carbon and iron conversion yield

## Abstract

The present work explores the role of the carbon source content and the Fe/C ratio on the synthesis of vertically aligned carbon nanotubes (VACNTs) by one-step aerosol-assisted CCVD operated at a medium temperature (615 °C) on aluminum substrates. The main objective was to overcome the limitations of VACNT growth, constituting a drawback for applications requiring thick VACNTs. By using acetylene as carbon feedstock and ferrocene as a catalyst precursor, we demonstrate that when acetylene content is reduced to 1.5 vol%, it is possible to grow VACNT carpets up to 700 µm thick while maintaining constant VACNT growth for a long duration (up to 160 min). The carbon conversion yield is significantly improved when the acetylene content reaches 1.5 vol%. The Al surface roughness also influences VACNT growth. An optimum Fe/C ratio of 0.8 wt.% coupled with a low acetylene content gives the highest growth rate (5.4 µm/min) ever reported for a thermal aerosol-assisted CCVD process operated at such a low temperature. The CNT number density can be controlled by varying the Fe/C ratio, enabling high density growth (e.g., 1.3 × 10^11^ CNT/cm^2^).

## 1. Introduction

Carbon nanotubes (CNTs) are attracting much interest due to their high thermal conductivity [1,2], electrical conductivity [3,4], mechanical strength and great flexibility [5,6,7]. They also show a high surface area and they can be chemically tuned via different chemical routes [8]. CNTs can be obtained by catalytic CVD (CCVD) in a vertically aligned configuration, forming carpets whose height can be controlled via various parameters (nature of the substrate, synthesis duration, composition of the reactive gas phase, etc.). Such architectures offer potential for many applications such as interconnects, sensors, electrodes for super-capacitors, etc. [9].

The relatively cheap and simple CCVD process, whether developed in one or two steps, has been up-scaled and is now operated at an industrial scale. However, reducing the energetic costs of the process while keeping good overall synthesis kinetics is a major current goal [10], since VACNT growth is thermally activated [11]. Moreover, the demand of VACNT applications in fields such as textiles, electronics or energy storage drives VACNT growth towards reduction of the process temperature, since substrates used in such fields are generally sensitive to heat [9,12].

Conventionally, VACNTs are synthesized by a two-step CCVD process. The first step involves the deposition of a catalytic layer made of metal oxides and its reduction, followed by a second step: introduction of a carbon precursor to grow VACNTs directly onto substrates of interest [8,12,13,14,15,16,17,18,19,20]. VACNT growth by a one-step CCVD process is a widely studied alternative since it offers the possibility to grow VACNTs through a continuous feeding of both the carbon source and the catalyst precursor, resulting in continuous VACNT growth at high temperatures [21,22,23].

Reducing the temperature (to around 600 °C) was investigated for two-step CCVD while keeping atmospheric pressure to maintain an industrially compatible process [12,24,25,26]. The challenges are focused on the enhancement of the industrial scalability of the process and on the possible synthesis of VACNT carpet directly on sensitive substrates such as aluminum (melting point: 660 °C), or thin aluminum-layer-coated substrates. As a drop in temperature will directly impact the growth rate [24,27], the CCVD process at low temperatures is most often assisted by a hot filament [28], a plasma [15,29], etc. However, such methods require additional energy sources, and this is limiting for industrial production.

Despite much progress, pure thermal two-step CCVD processes only result in a low VACNT growth rate (around 1–3 µm/min or less) [12,14,19,26]. Moreover, a decrease in growth rate is often observed or reported [12,19,20,25,26] at around 30 min synthesis, leading to a limitation in carpet thickness of around 100 µm with an average growth rate usually lower than 2 µm/min. It has been suggested that this limitation could originate from the reactive species’ diffusion into the carpet [30,31,32], or from the deactivation of the catalytic particle caused by carbon poisoning [24,33]. Because of the principle of the two-step process, the VACNT growth is performed with a limited amount of catalyst, increasing the chances of its deactivation for long-time synthesis. To overcome this issue, precise monitoring of the carbon precursor feedstock seems to be a key parameter to limit particle deactivation [34,35]. In addition, the introduction of oxygen was reported as beneficial for the growth of VACNTs, allowing carpet growth of up to a millimeter thickness [24,36,37]. Most of the oxygen sources used are H_2_O, CO_2_, CO, C_2_H_5_OH, O_2_ or air [14,24,25,36,37,38,39].

Another route involves continuous feeding of the catalyst precursor into the reactor. This one-step CCVD processes enables the constant renewing of the catalyst particles as reported at high temperature (850 °C) by Castro et al. [40]. However, in the literature, very few studies of VACNT synthesis with a single-step method are reported at a low temperature and atmospheric pressure [27,39,41,42,43,44]. Unlike two-step processes, here we can assume that particle deactivation will be limited. This is because the catalytic and carbon precursors are fed into the reactor during the whole synthesis, thus continuously bringing new fresh catalyst and keeping constant the catalyst to carbon mass ratio. However, in our recent study with an aerosol-assisted CCVD process operated at 615 °C on Al substrates, even if it was possible to significantly enhance the VACNT thickness (180 µm for an average growth rate of 2.3 µm/min), a carpet thickness limitation was still observed [27], most probably due to particle deactivation by catalyst poisoning [45]. Therefore, the VACNT thickness limitation remains an issue when CVD is operated at a low temperature, even for the one-step CCVD process.

The present work explores the effects of carbon source content and Fe/C ratio on VACNT growth and characteristics, with the aim of overcoming the VACNT growth limitation. Using a one-step aerosol-assisted CCVD process operated at 615 °C on Al substrates exhibiting various levels of surface roughness, and with C_2_H_2_ as carbon feedstock and ferrocene as the catalyst precursor, we demonstrate that it is possible to grow thick VACNT carpets of up to 700 µm and to enhance the growth rate up to 5.4 µm/min. The decrease of the acetylene content (factor 10) enables constant VACNT growth over a wide range of synthesis durations (up to 160 min), whereas the CNT number density can be controlled by the variation of the Fe/C ratio, enabling high densities (e.g., 1.3 × 10^11^ CNT/cm^2^). 

## 2. Materials and Methods

### 2.1. CCVD Synthesis of VACNTs

VACNTs are synthetized by a thermal CCVD process operated at low temperature (615 °C) and atmospheric pressure, and adapted from similar process described previously [27,46,47,48,49]. The aerosol-assisted CCVD is a one-step process where the reactor is supplied continuously and simultaneously by carbon (acetylene) and catalytic (ferrocene) precursors. Acetylene (99.6% purity, Messer Schweiz, Lenzburg, Switzerland) is used at various volume concentrations. Ferrocene (99% purity, Acros Organics, part of Thermofisher Scientific, Illkirch, France) is dissolved in toluene (99.9%, purity Analytical grade, Merck, Sigma-Aldrich Chemistry products, St. Quentin Fallavier, France) to reach the desired weight concentration (0.25 to 2.5 wt.%). It was previously shown in our lab [27] that toluene does not contribute significantly to VACNT growth at low temperature for our CVD process, hence justifying the use of acetylene.

The ferrocene–toluene solution is injected as an aerosol through an engine injection system (Qualiflow-Jipelec, Montpellier, France) and carried by a gas flow of argon (99.998% purity, Air Products, Aubervilliers, France) and hydrogen (99.9992% purity, Air Products, Aubervilliers, France). Hydrogen is used to enhance ferrocene decomposition and improve catalytic particle formation at such a low temperature (615 °C) [48]. The overall precursor and gas mixture is running through an evaporator heated at 250 °C in order to generate toluene/ferrocene vapors before entering the reactor. The quartz reactor with aluminum substrate inside is placed into a horizontal furnace (Carbolite Gero, Hope, UK, TZF 12/38/400) set at 615 °C which is the synthesis temperature involved in this study. After the injection period corresponding to the synthesis duration, the CVD reactor containing the grown VACNTs is cooled to room temperature under argon and hydrogen flow.

**Figure 1:**
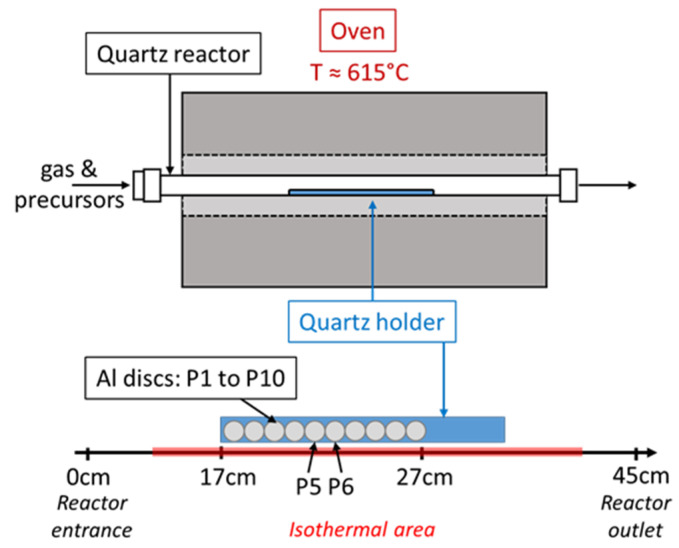
CCVD synthesis set-up and position of the Al substrates (discs) in the isothermal area. P5 is LG-Al substrate and P6 is HG-Al substrate.

Two grades of Al foils, a low-grade (LG-Al) and a high-grade (HG-Al) Al, exhibiting the same purity but different levels of surface roughness, are used and prepared according to a similar protocol. The objective is to identify a possible effect of the surface state on VACNT growth and quality. The low-grade Al (99.99% purity, 95 µm thickness, from SATMA PPC Company, Goncelin, France) is laminated and has a surface roughness of 400 ± 100 nm, whereas the high-grade Al (99.99% purity, 130 µm thickness, from Goodfellow, France) is polished and has a surface roughness of 50 ± 10 nm. Al discs, 10 mm in diameter, are first cleaned in acetone and ethanol. For all the experiments performed, ten discs, named P1 to P10, are placed on a quartz holder located in the isothermal area of the reactor (Figure 1). It is important to note that the position of the disc is constant despite the synthesis conditions performed in this study. VACNT growth occurs on the top face (named exposed face), and also the Al face in contact with the quartz holder (named hidden face) because the sealing between the Al discs and the quartz holder is not complete, thus enabling a partial reactive gas flow diffusion in the space between the Al substrate and the quartz holder. Most of the results presented in this paper are the ones obtained on the top face since it is the face directly exposed to the reactive gas flow.

### 2.2. Characterization

Characterization is mainly performed on the exposed face of the disc referenced “P5” for LG-Al and of the disc referenced “P6” for HG-Al, especially for SEM and TEM analysis. However, in order to acquire representative data for the mass and density of VACNTs, all the collected substrates (ten) placed in the isothermal area and covered with VACNTs on both faces are weighted. This allows us to calculate the carbon conversion yields on the overall substrate faces, which is representative of the CVD growth configuration.

The carpet thickness was measured using a Scanning Electron Microscope (SEM Carl Zeiss Ultra 55, Zeiss France, Rueil-Malmaison, France) through the analysis of the carpet cross-section on the exposed face of the Al disc referenced P5 (LG-Al) and P6 (HG-Al). Thickness measurement was performed on five different locations of the cross section: one at the extremities, two at the center and two in-between. Average thickness was then calculated.

To determine carpet chemical purity in terms of iron content, VACNT samples collected on the two faces of several discs of the same synthesis experiment were analyzed by thermogravimetric analysis (TGA). Such analyses (TGA, TA instrument TGA55) were performed under air, at a heating rate of 10 °C/min up to 700 °C during 10 min. During the heating treatment, CNT and catalyst oxidation occurs according to the reactions below, overall leading to a mass loss.

3C + 2O_2_ ⟶ 2CO + CO_2_ (1)

4Fe + 3O_2_ ⟶ 2Fe_2_O_3_ (2)

Oxidation of samples starts between 400 and 600 °C. After the TGA thermal cycle, a residual orange powder was collected in the alumina holder and has been identified as Fe_2_O_3_ by X-ray diffraction [46]. The mass of this residual oxide powder measured at the end of TGA analysis enables the calculation of the iron content in the initial sample according to chemical reaction (2). Typical TGA curve obtained for VACNT oxidation is shown in Figure S1. The iron conversion yield, which is the ratio between the iron content integrated inside the VACNT sample and the iron content injected from the ferrocene precursor, can thus be calculated. The carbon catalytic yield, defined as the ratio between the carbon content and the iron content inside the VACNT samples collected on both Al faces, is also determined.

Individual CNT morphology and size, as well as structure, are analyzed by Transmission Electron Microscopy (JEOL 2011, CIMEX platform, Ecole Polytechnique, Palaiseau, France). VACNTs are collected on the exposed Al face, and then sonicated in absolute ethanol (analytical grade). A droplet of the suspension is evaporated on a lacey carbon film deposited on a copper grid. Outer and inner CNT diameters are measured on fifty to a hundred CNTs for each analyzed sample.

## 3. Results

### 3.1. Synthesis Parameters Studied

Taking into account our previous results [27] and the state of the art in the field, the current study involves the variation of synthesis duration and contents in both carbon and catalyst precursors. The objective is to investigate low acetylene content while varying synthesis duration up to 160 min, which is a high duration compared to the literature. Their effects are studied both on the synthesis process and on the CNT growth and characteristics. Experiments with three different acetylene contents are performed. The highest acetylene content is similar to our previous study [27], i.e., 15% of the total flow rate. From this maximum value, acetylene content is reduced by three or by ten to investigate the effects of such a reduction on VACNT growth. As we already demonstrated that the Fe/C ratio has a strong influence on VACNT growth [27], the reduction of acetylene content is combined with a similar reduction of ferrocene concentration in toluene in order to keep the Fe/C mass ratio constant and lower than 1 wt.% [27], e.g., maintained in the (0.4–0.6) wt.% range in the present study.

Growth is performed on both LG-Al and HG-Al discs. Parameters are summarized in Table 1. The experiments using 15 vol% of acetylene up to 120 min duration are considered as the reference experiments since they are the reproduction of experiments performed by Nassoy et al. [27].

### 3.2. Synthesis and VACNT Overall Characteristics

**Figure 2:**
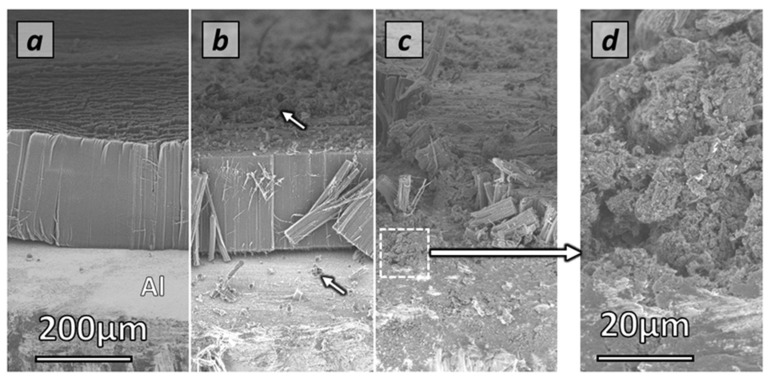
SEM micrographs on the exposed face showing: (**a**) typical homogeneous and clean VACNT carpet (low acetylene content, short synthesis duration); (**b**) typical homogeneous and unclean VACNT carpet (high acetylene content, long synthesis duration) showing carbon additional morphologies (see arrows) above and under the carpet; (**c**) typical inhomogeneous VACNT carpet showing a huge quantity of carbon by-products under the carpet (high acetylene content, long synthesis duration); (**d**) High magnification micrograph of carbon by-products observed in the white square area drawn on (**c**) micrograph (see arrow) and showing agglomerates of carbon particles.

After synthesis, whatever the conditions, all Al discs show a black or dark grey color on both faces. Some Al discs can occasionally bend or even roll up, especially for long synthesis durations (>80 min), whatever the acetylene content. Sample morphologies, directly observed by SEM on the cross-section of carpets grown on the exposed face of the Al substrates (Figure 1) show a vertical alignment of CNTs whose cleanliness depends on the synthesis conditions. It is noteworthy that very clean VACNT carpets are generally observed for synthesis performed at 5 and 1.5 vol% of acetylene (Figure 2a). However, especially for high acetylene content (15 vol%) and long synthesis duration, three additional morphologies possibly containing by-products can be observed: (i) craters on the Al substrate surface at the carpet base, and CNT entangled bundles at the top of the carpet (Figure 2b) as observed in our previous study [27], and (ii) agglomerated carbon particles at the carpet base (Figure 2c,d).

**Figure 3:**
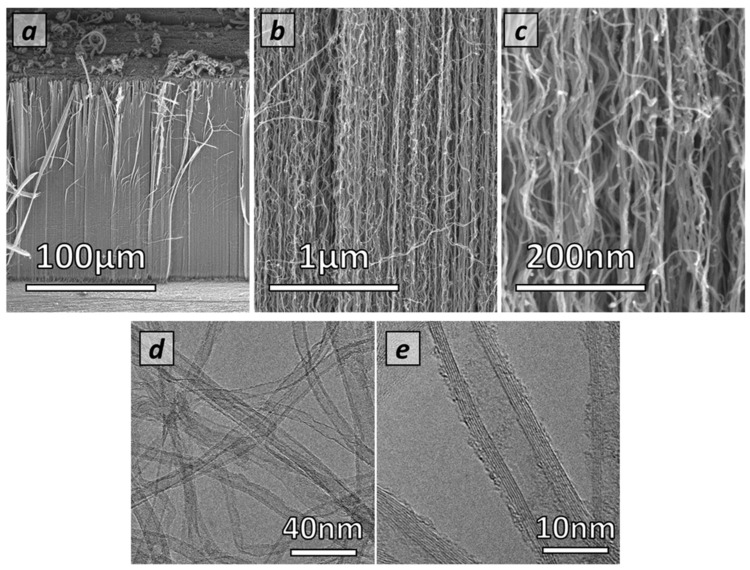
(**a**–**c**) typical SEM micrographs of clean VACNT carpet at different magnifications; (**d**,**e**) TEM and HRTEM micrographs of individual CNTs.

High magnification SEM observations of clean VACNT carpets, obtained from most of the synthesis conditions, indicate that the CNTs are well aligned and densely packed (Figure 3a–c). Individual CNT morphology, diameter and structure determined by TEM, indicate the formation of multi-walled CNTs (MWNTs) whose mean external diameters are in the [6,7,8,9,10,11,12] nm range, whatever the synthesis conditions (Figure 3d,e).

The iron content in VACNT samples grown on both substrate faces during intermediate duration (e.g., 80 min) and determined by TGA analysis is found to be 2.2 wt.% and 1.8 wt.% for high (15 vol%) and low (1.5 vol%) acetylene content, respectively. Taking into account these values, the iron conversion yield (ratio between the iron content integrated inside the VACNT sample and the iron content injected from the ferrocene precursor) is found to be 20.7 wt.% for high C_2_H_2_ content (15 vol%), while it reaches significantly higher values, 85.6 wt.%, for low C_2_H_2_ content (1.5 vol%).

The catalytic yield, calculated as the mass ratio between the carbon content and the Fe content inside the VACNT samples, gives an indication of the aptitude of catalytic nanoparticles to convert carbonaceous precursors into carbon solid materials. When VACNTs are clean (e.g., those obtained from 5 or 1.5 wt.% of acetylene) the catalytic yield is related directly to the formation of CNTs and not to the formation of a CNT/by-product mixture. For low C_2_H_2_ content, the catalytic yield is equal to 54, while, for comparison, for high C_2_H_2_ content it reaches a value of 44. This is consistent with the iron conversion yield which is higher for low C_2_H_2_ content, and could suggest that the catalytic efficiency of the Fe catalyst is higher when using low acetylene content.

The carbon conversion yield has been determined considering acetylene as the main carbon source since the synthesis is operated at quite a low temperature (Figure S2). It indicates that, whatever the synthesis duration, the carbon conversion yield is higher (around 20 wt.%) for 1.5 vol% of acetylene as compared to other acetylene contents (maximum 11 wt.% at 20 min duration and around 4.5 wt.% at 80 min duration for 15 vol% of acetylene). This result, combined with the results of the iron conversion yield, points out an effect of the lowest acetylene content (1.5 vol%), that will be discussed further in this paper.

### 3.3. Effect of Acetylene Content and Synthesis Duration on VACNT Carpet Thickness

**Figure 4:**
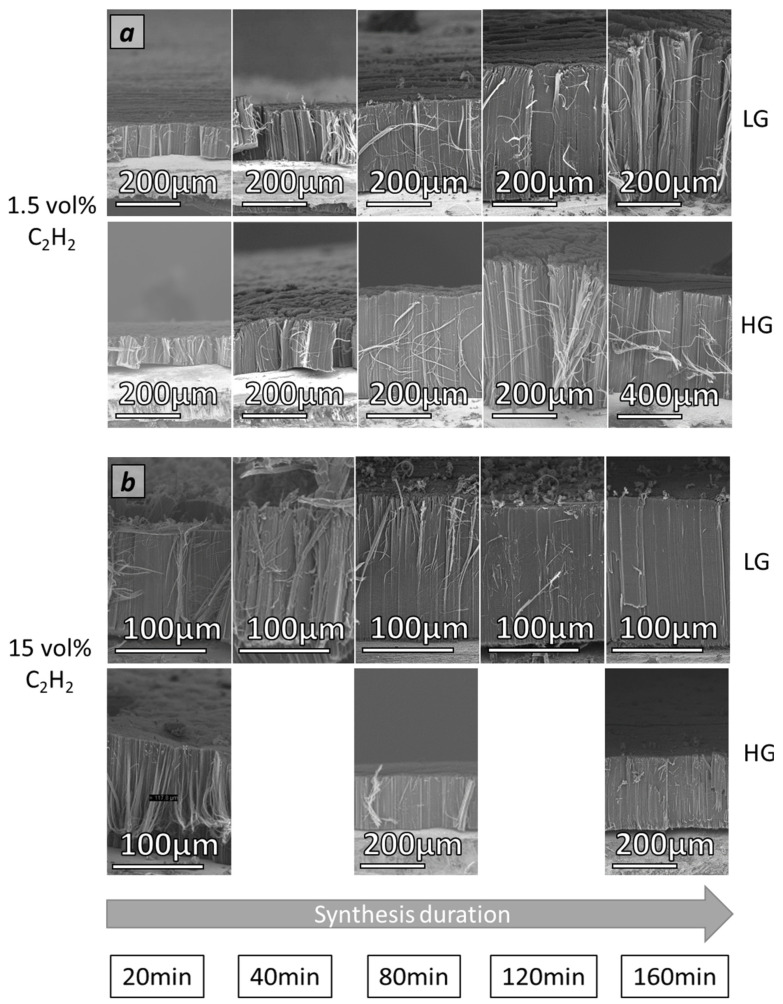
SEM micrographs of VACNT samples obtained on LG-Al and HG-Al substrates from (**a**) 1.5 vol% of C_2_H_2_ and (**b**) 15 vol% of C_2_H_2_ and for different synthesis durations.

All samples studied are obtained according to the conditions indicated in Table 1. Regarding their morphology and cleanliness, it is important to note that lowering the acetylene content (1.5 or 5 vol%) enables the generation of clean VACNT carpets with almost no by-products whatever the synthesis duration, as typically shown in Figure 2a and detailed for the different synthesis durations in Figure 4a (see Figure S3 for 5 vol% of acetylene). In comparison, higher acetylene content (15 vol%) combined with a long synthesis duration (from 40 min) involves the formation of VACNT carpets exhibiting carbon by-products on the top (CNT ropes, as shown in Figure 4b and at the base (CNT and carbon nanoparticle agglomerates) of VACNT carpets as typically shown in Figure 2c,d, which is consistent with results reported in the study of Nassoy et al. [27].

In addition, it is important to note that on HG-Al substrates, carpet cleanliness and general morphology are slightly improved as compared to LG-Al substrates. Indeed, carpets obtained on HG-Al with 1.5 vol% of acetylene are clean (Figure 4a) and easy to handle and process for further characterization.

**Figure 5:**
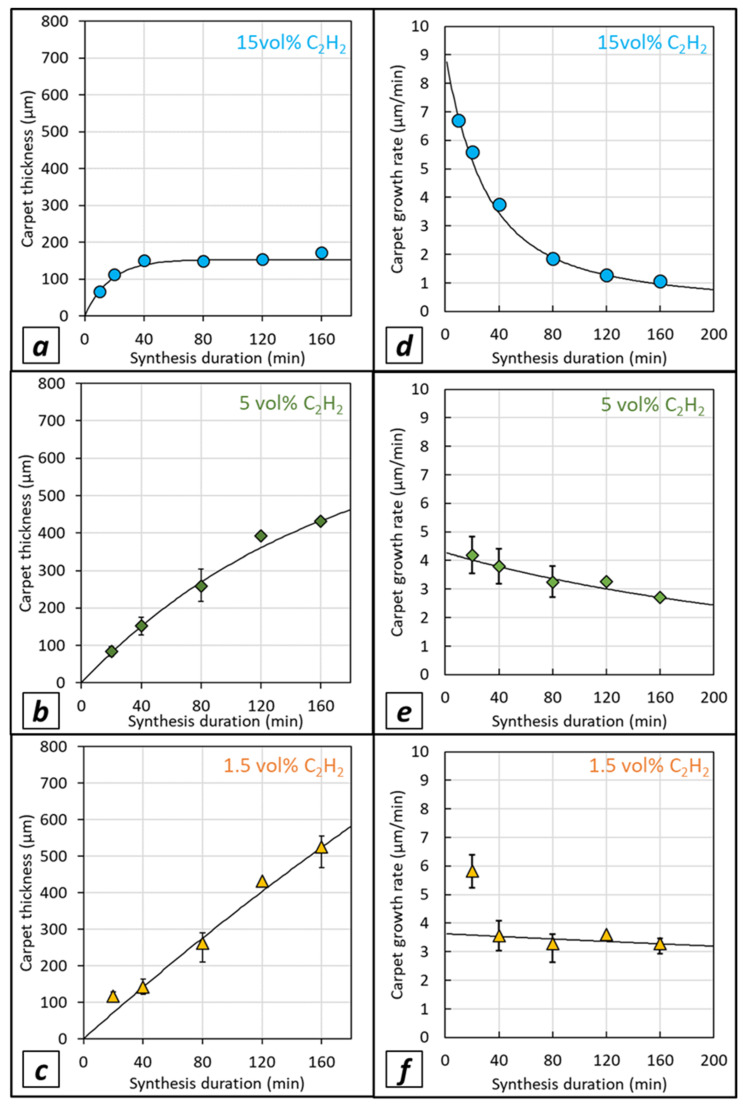
Carpet thickness versus synthesis duration on LG-Al substrates, for various acetylene contents (**a**) 15 vol%, (**b**) 5 vol% and (**c**) 1.5 vol%. Exponential model fittings are also plotted (plain black lines). Growth rate versus synthesis duration for various acetylene contents: (**d**) 15 vol%, (**e**) 5 vol% and (**f**) 1.5 vol%. Experimental dots correspond to the mean values calculated from 4 measurements performed on the cross-section of VACNTs observed on Al discs (P5 for LG-Al and P6 for HG-Al) for at least one synthesis experiment and up to three synthesis experiments performed in the same conditions. The variation ranges indicated around mean values correspond to the minimum and maximum of the mean value.

**Figure 6:**
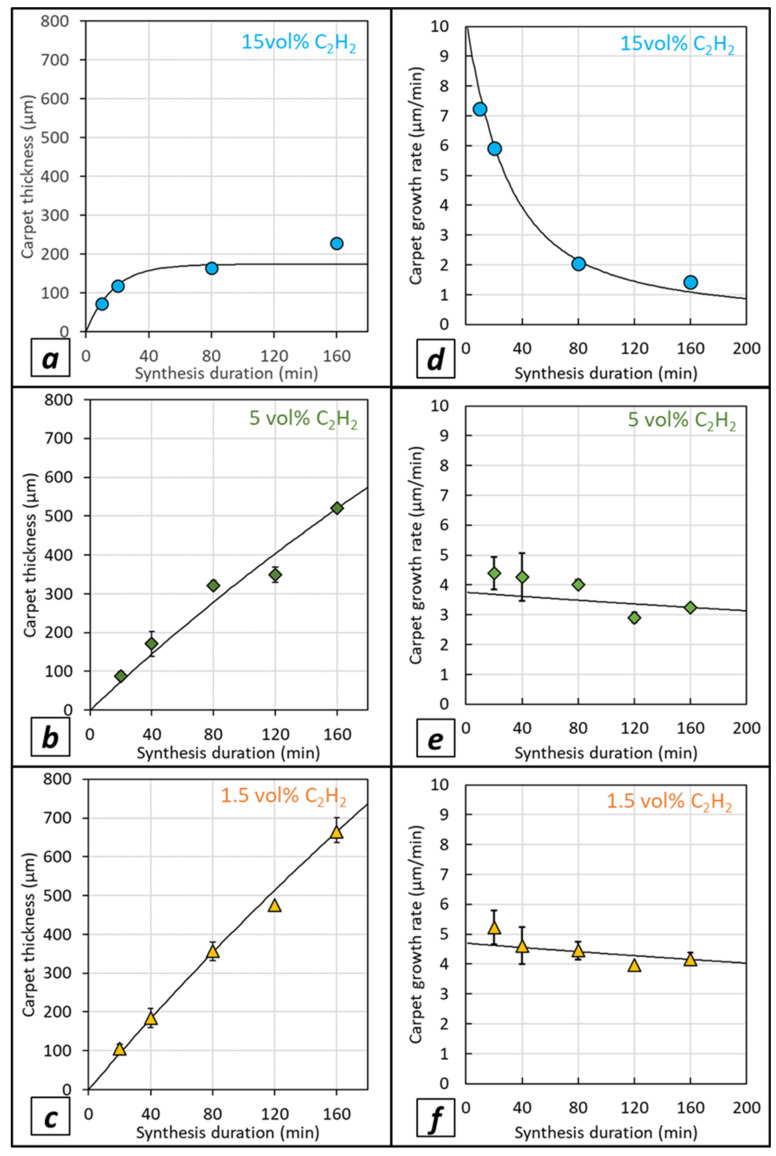
Carpet thickness versus synthesis duration on HG-Al substrates, for various acetylene volume contents in the gas phase: (**a**) 15 vol%, (**b**) 5 vol% and (**c**) 1.5 vol%. Exponential model fittings are also plotted (plain black lines). Growth rate versus synthesis duration for various acetylene volume contents in the gas phase: (**d**) 15 vol%, (**e**) 5 vol% and (**f**) 1.5 vol%. Experimental dots correspond to the mean values calculated from 4 measurements performed on the cross-section of VACNTs observed on Al discs (P5 for LG-Al and P6 for HG-Al) for at least one synthesis experiment and up to three synthesis experiments performed in the same conditions. The variation ranges indicated around mean values correspond to the minimum and maximum of the mean value.

VACNT carpet thickness (i.e., CNT length) versus synthesis duration for different acetylene contents is shown in Figure 5a–c for growth performed on LG-Al substrate and in Figure 6a–c for growth performed on HG-Al substrate. For 15 vol% of acetylene on LG-Al, as shown by Nassoy et al. [27], the carpet thickness increases progressively and then saturates from a 40 min synthesis duration, indicating that VACNT growth has stopped (see also Figure 4b). Lowering the acetylene content (≤5%) involves a strong difference in terms of VACNT thickness saturation, since in the synthesis duration range involved in this study (up to 160 min), the thickness progressively increases (Figure 5a–c), see also Figure 4a). Such a result demonstrates that lowering the acetylene content enables VACNT growth up to 160 min. The important consequence of such a behavior is that the VACNT thickness can reach up to 420 µm and 500 µm for acetylene content of 5 and 2.5 vol%, respectively (Figure 5b,c), which is 2.8 to 3.3 times higher than the thickness obtained (180 µm) for 15 vol% of acetylene.

Regarding the CNT growth rate (Figure 5d–f), for high acetylene content (15 vol%), a strong decrease from 7 to 1 µm/min is observed in the (20–160) min synthesis duration range studied, while using a lower acetylene content enables the drastic moderation of this growth rate decay. Indeed, it is noteworthy that lowering the acetylene content allows the maintenance of a growth rate of approximately 3 µm/min, even for longer synthesis durations of up to 160 min.

VACNT growth performed on HG-Al substrates (Figure 6a–c) indicates that carpet thickness reaches higher values as compared to LG-Al substrates (Figure 5b,c). While for high acetylene content the thicknesses vary in the same range as the ones obtained for LG-Al, e.g., up to approximatively 200 µm with a limitation from 40 min synthesis duration (Figure 5a); for HG-Al, carpet thickness can reach up to 700 µm in 160 min for 1.5 vol% of acetylene (Figure 6c). More importantly, the thickness increases almost linearly when synthesis duration increases and the thickness limitation is almost unobservable in this synthesis duration range, especially for synthesis performed with 1.5 wt.% of acetylene (Figure 6c). The trend regarding growth rate variation versus synthesis duration, with a strong decay occurring when high acetylene content is used, is similar for HG-Al as compared to LG-Al (Figure 5 and Figure 6a). However, for low acetylene content (1.5 and 5 vol%), the growth rate decreases only slightly (Figure 6e,f) as compared to the ones obtained on LG-Al substrates (Figure 5e,f), and the ultimate growth rate reaches 3 µm/min and 4 µm/min for 5 and 1.5 vol% of acetylene, respectively (Figure 6e,f), which is slightly higher as compared to LG-Al substrates, especially for 1.5 vol% of acetylene (Figure 5f).

The overall results indicate that lowering the acetylene content makes it possible to shift the thickness-limiting phenomenon over time and to reach high carpet thickness (e.g., 700 µm). Such a phenomenon is related to a constant growth rate (maximum 4 µm/min) maintained over a long duration, keeping high values for such a low synthesis operation temperature (e.g., 615 °C). This trend gets stronger as the acetylene content and/or the substrate surface roughness decreases. 

Many papers addressed this issue related to the limitation of carpet thickness over synthesis duration. The authors often consider feedstock diffusion through the carpet [45,50] or catalytic particle deactivation [27,33,51,52] as the reason for the growth rate decay over time, eventually leading to a total stop of CNT growth [12,24,27,33]. Our previous results [27] obtained for synthesis performed with 15 vol% of acetylene were successfully fitted by an exponential decay model (Equation (3)) describing the self-deactivation of catalytic particles [33,53]. In this model, the factor “*τ*” is the reaction time constant related to the catalytic particle lifetime and deactivation time, and “*max*” is the theoretical maximal thickness reached by the carpet, which is the product of *τ* by the initial growth rate γ_0_.

(3) $$y\left(t\right)=max\times \left(1-e^{-\frac{t}{\tau }}\right)$$

Results obtained in the present study for low acetylene content, either on LG- or HG-Al substrate, are fitted with the same model (fits in black plain lines in Figure 5 and Figure 6), and fitting factors obtained with a maximized R^2^ coefficient are presented in Table 2.

For both LG- and HG-Al substrates, the theoretical maximal carpet thickness (max) and the specific deactivation time (*τ*) are strongly increased when the acetylene content decreases from 15 vol% to 5 vol% and 1.5 vol%. This factor increase is important, as the acetylene content decreases, leading to a maximum thicknesses of 2.8 or 4.7 mm and a specific deactivation time of 770 or 1050 min for the lowest acetylene content (1.5 vol%) on LG- and HG-Al substrates, respectively (see Table 2). These values have to be compared to significantly lower values, e.g., 150 or 170 µm and 17 min for high acetylene content (15 vol%) on LG- and HG-Al substrates, respectively (see Table 2). These results strengthen the idea that a reduction of the acetylene content in the gas phase enables the prolonging of the catalytic particle lifetime and significantly helps to limit the VACNT carpet thickness saturation. Even more interesting, for the low acetylene contents in the synthesis duration range involved in this study (5 vol% and 1.5 vol%), the carpet thickness varies almost linearly with the synthesis duration, demonstrating that growth is maintained and suggesting that the catalytic particle is preserved from deactivation in such conditions, involving a constant growth at a rate of 3 to 5 µm/min which are high values for such low synthesis temperatures (615 °C). Using HG-Al substrates exhibiting a surface roughness approximatively ten times lower than that of the LG-Al substrates may affect the catalytic particle size or their arrangement, therefore involving a prolongation of their lifetime. 

### 3.4. Effect of Acetylene Content and Synthesis Duration on Individual CNT Morphology and Diameter

**Figure 7:**
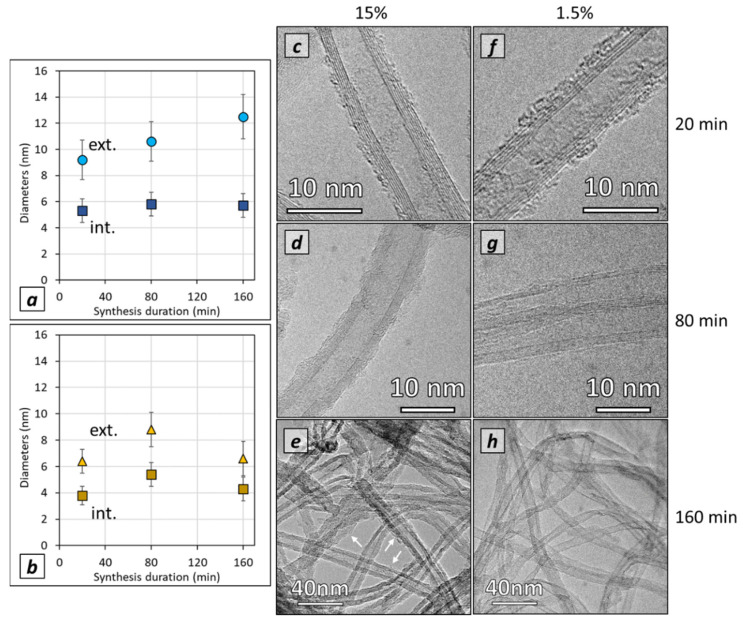
Average CNT external and internal diameter obtained on LG-Al substrates for: (**a**) high acetylene content (15 vol%), (**b**) low acetylene content (1.5 vol%). Measurements are performed by TEM on at least 100 CNTs observed; the mean values were calculated and reported together with the standard deviation (±2σ). TEM micrographs of CNT synthesized for (**c**–**e**) high acetylene content and (**f**–**h**) low acetylene content. Synthesis durations are (**c**,**f**) 20 min, (**d**,**g**) 80 min and (**e**,**h**) 160 min. White arrows indicate the presence of disorganized carbon.

Morphology and structure, as well as the external and internal diameters of individual CNTs grown on LG-Al substrates, are analyzed by TEM and HRTEM, especially for the highest and lowest acetylene contents. TEM observations (Figure 7) indicate that, whatever the synthesis conditions, multi-walled carbon nanotubes exhibiting some structural defects in their structures are observed. For high acetylene content, especially when the synthesis duration is high, it seems that some disorganized or amorphous carbon is occurring at the surface of the CNTs, whose occurrence is higher as compared to low acetylene content (Figure 7). The mean external diameter is found to continuously increase when the synthesis duration increases (Figure 7a), which could be explained by the additional carbon covering the CNTs. On the contrary, for low acetylene content (1.5 vol%) the external diameter is almost constant (ca. 7 nm) when synthesis duration increases up to 160 min (Figure 7b). For comparison, CNT diameters were measured on samples obtained on HG-Al substrates for an 80 min synthesis duration. A mean external diameter of ca. 7.5 to 8 nm is found, which is quite similar to the one found for CNTs on LG-Al substrate. The measurement of internal diameters of CNTs grown on LG-Al substrates indicates that whatever the synthesis duration, the mean internal diameter remains nearly constant, at ca. 6 nm and 5 nm for 15 vol% and 1.5 vol% of acetylene, respectively (Figure 7a,b). Using HG-Al substrate does not involve any change in the internal diameter, which remains similar to that of the CNTs grown on LG-Al substrates. These results and trends (Figure 7) are consistent with the external and internal diameter distribution (Figure S4).

The number of walls (Figure S5), calculated from the external and internal diameter, increases when the synthesis duration increases with a high acetylene content on LG-Al substrates (i.e., from 7 ± 1 walls at 20 min to 11 ± 2 walls at 160 min) while it remains constant and lower (around 5 ± 1 walls) for CNTs synthesized at a low acetylene content (1.5 vol%). RAMAN spectroscopy was performed on VACNT carpet samples. The D band over G band intensity (ID/IG ratio) average was found to be 1.6 whatever the synthesis conditions. This is rather common for low-temperature-grown VACNTs [25,27,30], and attests to the presence of defects in CNT structure. Typical RAMAN spectrum for low acetylene content and 20 min synthesis duration is shown in Figure S6.

### 3.5. Effect of Acetylene Content and Synthesis Duration on Carpet Density

**Figure 8:**
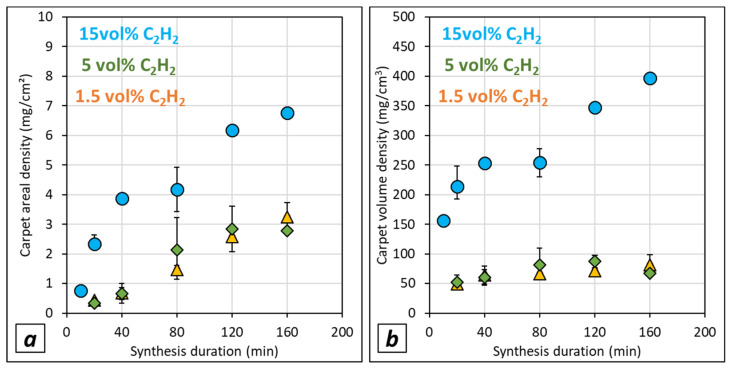
Carpet density calculated from the sample weights on the two faces of the LG-Al substrates (P5) after synthesis from various acetylene contents (15 vol% blue dots, 5% vol% green diamond, 1.5 vol% yellow triangles) performed in the (20–160) min duration range: (**a**) areal density, (**b**) volume density, both drawn versus synthesis duration. When indicated, variation range around the mean value is calculated from at least 2 synthesis experiments performed in the same conditions, and corresponds to the minimum and maximum of the mean value.

Both areal and volume density are calculated from the VACNT sample weights collected on the two substrate faces in order to acquire global density values, and are presented versus synthesis duration in Figure 8 for samples grown on LG-Al substrates. Whatever the acetylene content, the areal density increases when the synthesis duration increases, which is in good agreement with the trends observed for VACNT thickness versus synthesis duration, apart from the 15 vol% of acetylene, for which carpet thickness saturates from 40 min synthesis duration. In this case, the increase of areal and volume density could be due to the occurrence of additional carbon morphologies as shown in the previous sections. The main difference between samples obtained at 15 and 5 or 1.5 vol% of acetylene is that the areal density values are lower for synthesis performed with low acetylene content (5 and 1.5 vol%). The same trends are observed for the volume density even if, for 15 vol% of acetylene, it increases more drastically with synthesis duration (from 150 to 400 mg/cm^3^) than for 1.5 and 5 vol% of acetylene, which display variations that are quite similar (from 50 to 100 mg/cm^3^). Such a difference between samples obtained with 15 vol% of C_2_H_2_ and 5 or 1.5 vol% of C_2_H_2_ is most likely related to the ferrocene content in toluene which has been reduced to keep constant the Fe/C ratio while decreasing the acetylene content. Indeed taking into account the formation of catalyst nanoparticles through a homogeneous nucleation process as reported by Castro et al. [47], ferrocene concentration contributes to the control of the catalyst nanoparticle size and density as mentioned by Charon et al. [49]. However, as the ferrocene concentration is lower for 1.5 vol% than for 5 vol% of acetylene, the volume density should be lower for 1.5 vol%, which is not verified in Figure 8. This striking result will be discussed in the following section.

One must also note that the same trends are also obtained when the mass of the VACNT sample is only considered on the exposed face (Figure S7). However, a higher dispersion occurs, most likely due to the preparation of the samples involving the stripping of VACNT carpets grown on the hidden face.

In the present study, the overall results shown previously indicate that operating AACCVD with a low acetylene content gives strong advantages in terms of carpet growth kinetics, and consequently enables the removal of the thickness limitation phenomenon in the synthesis duration range examined. In addition, lowering the acetylene content does not affect the carpet quality since carpets without additional morphologies are mainly obtained throughout the whole synthesis duration range. However, the volume density is lowered. As this study have been performed with a constant Fe/C ratio of ca. 0.4 wt.%, it is thus important to complete this work by studying the variation of Fe/C ratio at the constant acetylene content of 1.5 vol%, which gives the best results in term of constant growth rate over the synthesis duration. 

### 3.6. Effect of Fe/C Ratio on VACNT Growth and Carpet Density

**Figure 9:**
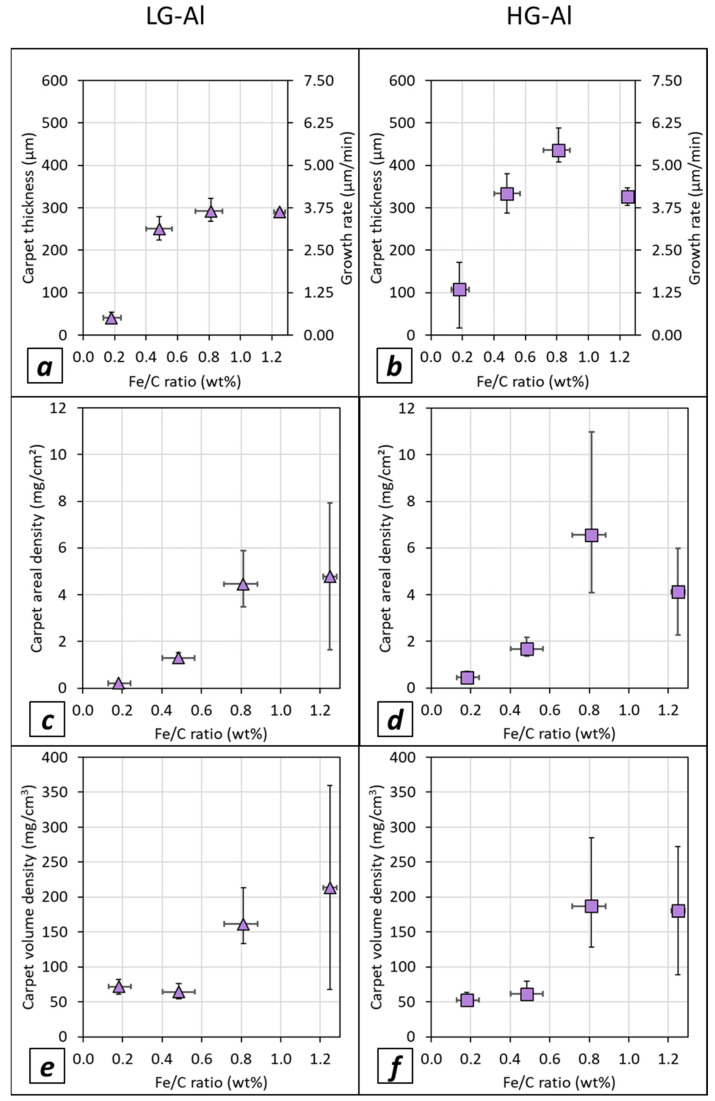
Carpet thickness, areal density and volume density versus Fe/C ratio for synthesis performed with 1.5 vol% of acetylene during 80 min: (**a**–**c**) on LG-Al substrate; (**d**–**f**) on HG-Al substrate. The variation range on the horizontal axis corresponds to the minimum and maximum values investigated around the mean value, and the variation range on the vertical axis corresponds to the minimum and maximum of the mean values obtained.

The variation of the Fe/C ratio on VACNT growth is studied in the (0.1–1.3) wt.% range on both LG (Figure 9a–c) and HG (Figure 9d,f) Al substrates. Symbols (triangles or squares) are VACNT thickness averages obtained from two or three synthesis experiments. Horizontal variation ranges depict experimental variation of Fe/C. Syntheses are performed with 1.5 vol% of acetylene during 80 min which corresponds to a fairly linear growth regime for Fe/C of 0.4 wt.%. In these conditions, the carbon conversion yield, considering the acetylene as the carbon source, increases when the Fe/C ratio increases, and reaches ca. 60 wt.% when Fe/C ratio is greater than or equal to 0.8 wt.% (see Figure S8), which is three times higher than the one reported in the previous section for the 0.4 wt.% Fe/C ratio (20 wt.%).

Regarding the quality of the samples obtained, some Al discs supporting the nanotubes can become deformed and fold back on themselves. This deformation phenomenon occurs particularly when the Fe/C ratio is higher than 0.6 wt.% and becomes strong when it reaches values of ca. 1.2 wt.%. However, in the (0.6–0.8) wt.% Fe/C ratio, the samples are still easy to handle and exploitable since only few of them are deformed.

For both substrates, the variation of carpet thickness and CNT growth rate is quite similar: the height increases continuously up to a Fe/C limit value and then tends to reach almost a plateau (Figure 9a,b); see also Figure S9 showing SEM observations of VACNTs. It is important to note that VACNT thicknesses at the plateau, and thus growth rates, are higher for the HG-Al substrate (reaching 450 µm and ca. 6.25 µm/min) in comparison to the LG-Al substrate (300 µm and 4 µm/min). These results confirm that surface roughness plays a role in carbon nanotube growth even for various Fe/C ratios. 

The areal and volume densities (Figure 9c–f) have similar trends whatever the type of substrate considered. Indeed, for the areal density, a slight increase is observed in the (0.1–0.5) wt.% Fe/C range, while a sudden increase occurs from a 0.5 wt.% Fe/C ratio. For the volume density, similar trends are obtained, apart from the (0.1–0.5) wt.% Fe/C range where the volume density is almost constant. Then, suddenly, it increases from a 0.5 wt.% Fe/C ratio reaching the highest values of ca. 200 mg/cm^3^ for both LG- and HG-Al substrates. 

**Figure 10:**
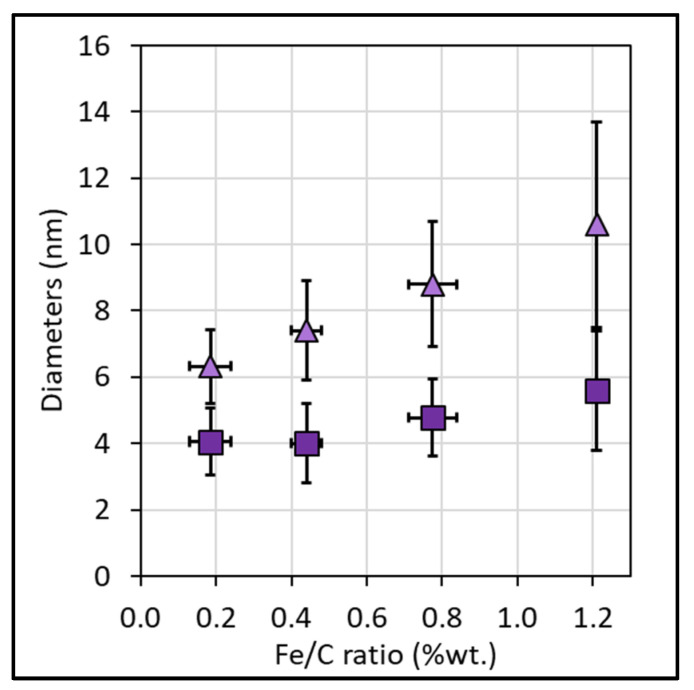
Average CNT external (triangle) and internal (square) diameter obtained on HG-Al substrates. Measurements are performed by TEM on at least 100 CNT observed, the mean values are calculated and reported together with the standard deviation (±2σ).

CNT external diameters (Figure 10), measured by TEM on CNT grown on HG-Al substrate, increase from 6.3 ± 1.1 nm to 10.6 ± 3.1 nm when the Fe/C ratio increases from 0.2 to 1.2 wt.%, while the internal diameter only slightly varies (from 4.1 ± 1.0 nm at Fe/C of 0.2% to 5.6 ± 1.8 nm at Fe/C of 1.21%). In addition, the external diameter distribution is enlarged when Fe/C increases, especially from 0.8 wt.% (see Figure S10). Taking into account that the samples are mainly composed of aligned CNTs in the overall Fe/C range studied, these results indicate that the number of graphene walls increases when the Fe/C ratio increases. The variation is included in the (4–8) wall range with quite a high dispersion (between 4 to 11 walls) when the Fe/C ratio reaches 1.2 wt.% (see Figure S11).

**Figure 11:**
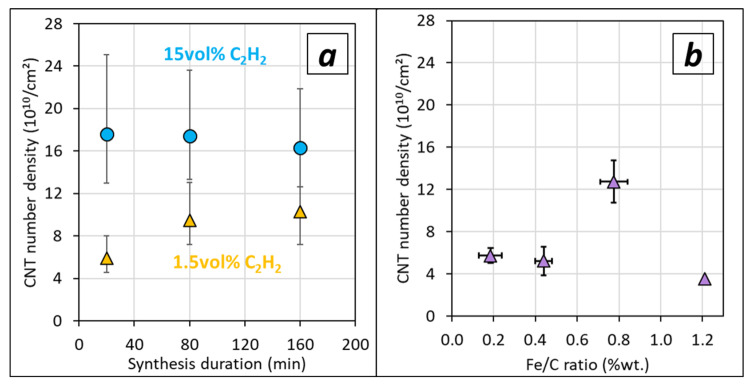
CNT areal number density calculated on samples obtained on HG-Al substrates versus (**a**) synthesis duration for high (15 vol%, blue circle) and low (1.5 vol%, orange triangle) acetylene content, and (**b**) Fe/C ratio for low acetylene content and 80 min synthesis duration. Experimental dots correspond to mean values calculated from mean diameters and lengths, and variation ranges correspond to the standard deviations (±2σ).

Taking into account the diameter values and the volume density, it is possible to calculate the CNT areal number density for HG-Al substrate for different acetylene contents (Figure 11a). When synthesis duration increases for a constant Fe/C ratio of 0.4, the CNT density slightly decreases from 1.8 to 1.6 × 10^11^ CNT/cm^2^ for high acetylene content, while it slightly increases and stays lower (ca. 9 × 10^10^ CNT/cm^2^) for low acetylene content. The variation of Fe/C ratio at low C_2_H_2_ content (synthesis duration 80 min) demonstrates that the number density is reasonably maintained (ca. 5 × 10^10^ CNT/cm^2^) in the (0.1–0.5) wt.% Fe/C range, but increases by a factor of 2 to 3 when the Fe/C ratio increases to 0.8, then reaches values (1.3 × 10^11^ CNT/cm^2^) almost similar to the ones obtained (1.8 × 10^11^ CNT/cm^2^) for samples synthesized with a high acetylene content for the same duration (80 min).

## 4. Result Summary and Discussion

**Figure 12:**
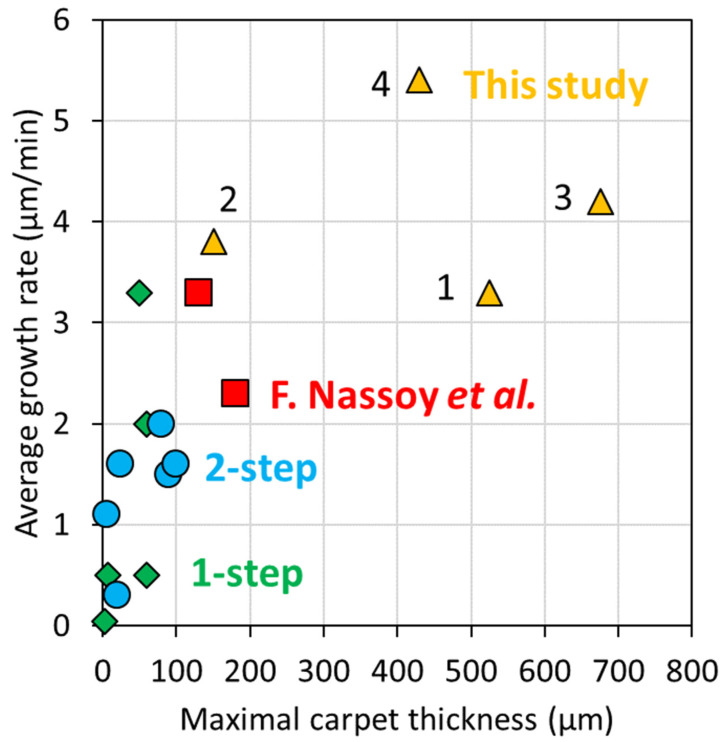
Average growth rate compared to maximal carpet thickness achieved, reported for low temperature one-step CVD process (green diamond), two-step CVD process (blue circles), and a study performed by F. Nassoy et al. (red squares). For comparison, present results obtained in our study are also shown (yellow triangles). Numbers indicate different synthesis conditions for VACNT growth, which are low acetylene content on low grade Al (1), high acetylene content on low grade Al (2), low acetylene content on high grade Al (3) and low acetylene content on high grade Al with enhanced Fe/C ratio of 0.8% (4).

Results regarding the study of the variation of the acetylene content, at a constant Fe/C ratio and in a large synthesis duration range, indicate that the decrease of acetylene content down to a factor of ten enables a strong enhancement of VACNT growth. Indeed, as compared to 15 vol% of acetylene, for low grade aluminum substrate, the VACNT thickness is 2.8 to 3.3 times higher when the acetylene content is lowered down to 5 and 1.5 vol%, respectively. The maximum thickness reached increases as the acetylene content decreases, e.g., 425 µm and 525 µm (dot N°1 in Figure 12) for an acetylene content of 5 and 1.5 vol%, respectively, as compared to 180 µm for 15 vol% of acetylene (dot N°2 in Figure 12). More interestingly, for these low acetylene contents, VACNT growth is maintained in the whole synthesis duration range, up to 160 min, contrary to VACNT growth saturation, occurring from 40 min when 15 vol% of acetylene is used. Indeed, while the growth rate decreases drastically for 15 vol% of acetylene (from 7 to 1 µm/min), using a low acetylene content involves a progressive VACNT thickness raise when the synthesis duration increases up to 160 min, and a mean growth rate conservation even for long synthesis durations (ca. 3 µm/min for both low acetylene contents). 

The same trends are obtained for high grade Al substrates, and furthermore, the effects are enhanced as compared to the ones observed on low grade Al substrates. Indeed, the maximum thickness reaches significantly higher values, ca. 700 µm in 160 min for 1.5 vol% of acetylene (dot N°3 in Figure 12), and the growth rate is maintained constant and higher in the whole synthesis duration range (3 and 4 µm/min for 5 and 1.5 vol% of acetylene, respectively).

These results were compared to literature related to thermal CCVD growth of VACNTs at ca. 600 °C, and to the best of our knowledge, VACNT growth rates and thicknesses obtained in this study are the highest ever reached on Al substrates (Figure 12 and Table 3). Decreasing the acetylene content enables us to maintain VACNT growth for longer synthesis durations up to the maximum investigated in this study (160 min), which is significantly longer than the duration ranges investigated in similar studies (see references in Table 3).

The beneficial effect of the lowest acetylene content (1.5 vol%) is further improved when the iron-based catalyst precursor content is adjusted. Effectively, an optimum Fe/C ratio of 0.8 wt.% was determined, generating VACNT carpets as high as 430 µm for only an 80 min synthesis duration, and corresponding to a mean growth rate of 5.4 µm/min (dot N°4 in Figure 12). Such a growth rate is the maximum ever reported in the literature for thermal CCVD processes (Figure 12 and Table 3). 

Overall, decreasing the acetylene content down to 1.5 vol% enables us to reduce drastically, or even suppress (in the case of the HG-Al substrate), the VACNT growth limitation for long synthesis durations up to 160 min, contrary to the highest acetylene content (15 vol%) involving a growth limitation from 40 min synthesis duration. Taking into account our previous results [27] and the literature [33,50,51], it was assumed that the origin of such a limitation is due to the deactivation of the catalyst particle since the experimental results obtained for 15 vol% of acetylene were successfully fitted by an exponential decay model describing the self-deactivation of catalytic particles [33,53]. The same model was tested on the present results: it indicates that the maximum reachable thickness and the specific deactivation time of the catalyst particle are as high as the acetylene content is low, and are even higher when an HG-Al substrate is used. However, even if the fitting curves are acceptable, it is noteworthy that in the (0–160) min synthesis duration range and for the lowest acetylene content, a quasi-linear increase of the VACNT thickness occurs, especially for an HG-Al substrate. Therefore, the thickness limitation is almost unobservable in this large synthesis duration range. This strong result strengthens the idea that high carbon feeding of the reactive gaseous phase contributes, even for the one-step CCVD process, to a rapid deactivation of the catalyst particle as reported mainly for the two-step CVD processes [24,27,33,34]. In addition, according to the present results, the catalyst feeding is also an important parameter to control the VACNT growth dynamic, since for low acetylene content, increasing the Fe/C ratio enables us to reach higher mean growth rates on both grade Al substrates. Even more interestingly, this resulting growth enhancement is as high as the surface roughness of the Al substrate is low, suggesting that the growth or the agglomeration of catalyst nanoparticles are favored in the grooves [18,26,39,54], involving a change in their behavior, especially the formation of amorphous carbon or carbon onions, which contributes to catalyst nanoparticle poisoning and eventually to their deactivation. 

The growth improvement, higher for the lowest acetylene content, can be related to the iron and carbon conversion yields. For short synthesis duration (e.g., 20 min), the carbon conversion yield at low acetylene content (1.5 vol%) is almost two times higher than the one at 15 vol% C_2_H_2_. For long duration (e.g., 80 min), the carbon conversion yield drops for 15 vol% while it remains the same for 1.5 vol% and is then four times higher. Interestingly, for synthesis performed during 80 min (e.g., in the height saturation regime for 15 vol% of acetylene while in the linear regime for 1.5 vol% of acetylene), the iron conversion yield for 1.5 vol% of acetylene is also four times higher than the one obtained for 15 vol% of acetylene. The decrease of the acetylene content is performed together with a decrease of the ferrocene content to keep a constant Fe/C ratio (0.4 wt.%). Therefore, such a high iron conversion yield suggests a continuous incorporation of iron into VACNTs throughout the synthesis when the ferrocene concentration is lowered and/or when the acetylene content is reduced, leading to synthesis without saturation. This result strongly suggests that, while keeping a constant Fe/C, ferrocene does not behave similarly according to the acetylene content: a better catalytic activity is demonstrated when the acetylene is significantly lowered. This could be explained by a higher ferrocene decomposition rate. Indeed, ferrocene decomposition is enhanced by hydrogen [48], and when a low ferrocene content is used, the hydrogen content is higher relative to the ferrocene concentration, thus enabling an enhancement of the ferrocene decomposition and subsequently of the iron incorporation. Another hypothesis could rely on the acetylene, which could affect the catalyst activation degree as was recently reported by Khabushev et al. [55] for synthesis performed by a floating CVD process operated at 1100 °C using ethylene and toluene/ferrocene. Since acetylene and ethylene are two carbon molecules whose decomposition behaves quite similarly, using such low acetylene content could generate an increase in the fraction of the active Fe particles together with the CNT growth rate. Even if the operating temperatures are very different (615 °C in our study), we find it interesting to mention the similarities between our results and the trends reported by Khabushev et al. [55]. Effectively, they found a threshold ethylene content (0.15 vol%) below which beneficial effects on catalyst activation degree together with CNT growth rate were found, while above this content, a deterioration of the synthesis productivity was reported and explained by the catalyst poisoning by carbon. In our study, at low acetylene content (1.5 vol%), increasing the Fe/C ratio to 0.8 wt.% and above enabled a significant enhancement of the carbon conversion yield (three times higher than the one obtained for Fe/C of 0.4 wt.%), indicating an interplay effect of acetylene and ferrocene concentrations that could justify the enhancement observed in the CNT growth rate, since in these conditions, VACNTs do not contain any additional carbon morphology. In addition, the significant increase occurring on the carbon conversion yield for synthesis operated at 5 vol% to 1.5 vol% of acetylene, while keeping a constant Fe/C ratio (0.4 wt.%) (Figure S2), strengthens the hypothesis of such a synergetic effect. 

The Fe/C ratio plays also an important role on nanotube mean diameters as well as on VACNT density. Indeed, when Fe/C increases from 0.2 to 1.2 wt.%, the internal and external diameter progressively increases and the amplitude of the gain for the external diameter reaches almost a factor of two for VACNT grown on an HG-Al substrate. In addition, this gain occurs together with an enlargement of the distribution in diameter and number of walls. Since it is commonly assumed that the external diameter is representative of the catalyst particle diameter [56,57,58], our results strongly suggest that catalyst particle size increases when the Fe/C ratio increases. Meanwhile, this is in contradiction with the study of Castro et al., which focused on an aerosol-assisted CCVD process operated at 850 °C on silicon or quartz substrates, and indicated that the formation of catalyst nanoparticles follows a homogeneous nucleation phenomenon in the gas phase, therefore generating the formation of smaller particles when the ferrocene content is increased [47]. However, in the present study, the nature of the substrate (Al) and the operating temperature (615 °C) are different, involving certainly a different mobility of catalyst nanoparticles on the ductile aluminium substrate, and therefore a particle coarsening that is as high as the density of catalyst nanoparticles formed in the gas phase is important (case of high Fe/C ratio). This particle coarsening could have played a limiting role on the VACNT growth rate, which is not observed probably due to the synergetic effect between acetylene and ferrocene concentrations on the catalyst activation degree. Besides, the areal and volume density also increased, even if the gain was not progressive but occurred mainly between Fe/C of 0.5 wt.% and Fe/C of 0.8 wt.%. This suggests that the density of catalyst nanoparticles together with the catalyst activation degree (as discussed above) are increased when the Fe/C ratio increases from 0.5 wt.%. Taking into account only the homogeneous nucleation phenomenon, for a constant Fe/C ratio (0.4 wt.%), since ferrocene concentration is higher for 5 vol% of acetylene, the volume density of VACNT samples obtained should be higher as compared to the ones obtained from 1.5 vol% of acetylene. However, volume density is similar for both syntheses, strengthening the hypothesis of a synergistic effect between acetylene and ferrocene concentrations [55].

The resulting CNT density depends on the trends mentioned above. While decreasing the acetylene content is beneficial for VACNT growth, especially at 1.5 vol% of acetylene, the volume density and the CNT number density can be adjusted through the adjustment of the Fe/C ratio. Indeed, for the lowest acetylene content (1.5 vol%) involving the best growth, the CNT number density increases by a factor of two to three when the Fe/C ratio increases to 0.8. The resulting value (1.3 × 10^11^ CNT/cm^2^) is almost similar to the ones obtained (1.8 × 10^11^ CNT/cm^2^) for samples synthesized with high acetylene content and correctly compares with our previous results obtained at a high acetylene content (15 vol%) [27].

## 5. Conclusions

In summary, we have investigated the role of the carbon source content and of the Fe/C ratio (depending on catalyst precursor and carbon source content) in the reactive gas phase on the synthesis of VACNTs by a thermal aerosol-assisted CCVD process operated at medium temperature (615 °C). A systematic study over a wide synthesis duration range (up to 160 min) was performed using two grades of Al substrates differentiated by their surface roughness. The acetylene content and the Fe/C weight ratio were varied. The main purpose was to enhance VACNT growth at such a relatively low temperature and to investigate the possibility of delaying the catalyst deactivation phenomenon observed previously for synthesis performed with 15 vol% of acetylene [27].

The present results demonstrate that significantly reducing the acetylene content down to 1.5 vol% is even able to suppress completely the VACNT thickness limitation, and therefore to strongly limit the catalyst deactivation phenomenon. In addition, an optimum Fe/C ratio of 0.8 wt.% coupled with this low acetylene content involves the highest growth rate (5.4 µm/min) ever reported for such a thermal aerosol-assisted CCVD process operated at such a low temperature. The carbon conversion yield is significantly improved when the acetylene content reaches 1.5 vol%, while it remains quite similar for 5 and 15 vol% of acetylene, supporting the hypothesis of a joint effect of the acetylene content and the ferrocene concentration enhancing the catalyst activation degree and the CNT growth rate. 

The roughness of the Al substrate is another important parameter influencing VACNT growth rate. A high roughness involves a lower CNT growth, most probably because the catalyst particle configurations are changed inside the grooves, forming agglomerates which are less effective for CNT growth.

Overall, this study demonstrates that a significant decrease of the acetylene content (factor 10) makes it possible to maintain constant VACNT growth for long synthesis times (up to 160 min). VACNT carpets exhibiting thicknesses up to 700 µm are obtained with quite a high growth rate (4.2 µm/min) on smooth Al substrates through an aerosol-assisted synthesis process operated at 615 °C without any other assistance (plasma, hot filament, …). The carpets remain clean and the CNT number density can reach high values (e.g., 1.3 × 10^11^ CNT/cm^2^). 

## Figures and Tables

**Table 1:** Synthesis durations and acetylene concentrations used for the different experiments performed. LG-Al and HG-Al stand for Low-Grade and High-Grade aluminum substrate, respectively.

Synthesis Duration	Acetylene Content (vol%)	
(min)	15%	5% & 1.5%
20	LG-Al&HG-Al	LG-Al&HG-Al
40	LG-Al	LG-Al&HG-Al
80	LG-Al&HG-Al	LG-Al&HG-Al
120	LG-Al	LG-Al&HG-Al
160	LG-Al&HG-Al	LG-Al&HG-Al

**Table 2:** Fitting factors obtained with Equation (3) and results obtained for various acetylene volume contents on: (left) LG-Al substrate, and (right) HG-Al substrate.

Fitting Factors	Acetylene Content (LG-Al)			Acetylene Content (HG-Al)		
	15%	5%	1.50%	15%	5%	1.50%
Maximal thickness (µm)	153	684	2790	174	2019	4700
Specific deactivation time (min)	17	160	769	17	538	1050
Initial growth rate (µm/min)	9.0	4.3	3.6	10.2	3.8	4.5
R^2^ coefficient	0.986	0.994	0.988	0.782	0.974	0.995

**Table 3:** State of the art VACNT growth results for low temperature CVD process, mostly performed with iron catalytic precursors and aluminum-based substrates, giving rise to Figure 12. Our results obtained in the present study are also reported at the end of the table.

**State of the Art**	**CVD Process**	**Max. Thick. (µm)**	**Avg. Growth Rate (µm/min)**	**Substrate**	**Cat. Precursor**	**Carb. Precursor**
Andrews1999	1-step	60	0.5	Quartz	Ferrocene	Xylene
Kuo2006	1-step	7	0.5	Si	Fe(CO)_5_	Acetylene
Lou2013	1-step	60	2	Al	Ferrocene	Ethanol
Arcila-Velez2014	1-step	50	3.3	Al	Ferrocene	Acetylene
Fleming2019	1-step	2	0.04	Al_2_O_3_	none	Acetylene
Nassoy2019	1-step	130	3.3	Al	Ferrocene	Acetylene
Nassoy2019 Seq.	1-step	180	2.3	Al	Ferrocene	Acetylene
Du2020	1-step	33	0.5	Al	Ferrocene	Ethanol
Yoshikawa2008	2-step	90	1.5	Al	Fe + Co	Ethanol
Reit2013	2-step	5.3	1.1	Al-NiTiAl	Fe	Acetylene
Dörfler2013	2-step	80	2	Al	Fe + Co	Ethylene
Gao2015	2-step	100	1.6	Si-Al	Fe	Acetylene
Almkhelfe2016	2-step	20	0.3	Si-Al_2_O_3_	Fe	Fischer-Tropsch gaseous mix
Szabo2017	2-step	24	1.6	Al	Fe + Co	Ethylene
**This Work**	**CVD Process**	**Max. Thick. (µm)**	**Avg. Growth Rate (µm/min)**	**Substrate**	**Cat. Precursor**	**Carb. Precursor**
Combrisson LG15% and Fe/C of 0.4%	1-step	150	3.8	Al	Ferrocene	Acetylene
Combrisson LG1.5% and Fe/C of 0.4%	1-step	525	3.3	Al	Ferrocene	Acetylene
Combrisson HG1.5% and Fe/C of 0.4%	1-step	675	4.2	Al	Ferrocene	Acetylene
Combrisson HG1.5% and Fe/C of 0.8%	1-step	430	5.4	Al	Ferrocene	Acetylene

## Data Availability

Data presented in this article are available at request from the corresponding author.

## Supplementary Materials

The following supporting information can be downloaded at: https://www.mdpi.com/article/10.3390/nano12142338/s1, Figure S1. Typical thermo-gravimetric analysis under air indicating the oxidation of VACNT. Temperature, represented by the red curve, rises from room temperature to 700 °C at 10 °C/min. Sample mass during TGA is represented by the blue curve; Figure S2. (a) Carbon conversion yield from acetylene calculated by dividing CNTs total mass collected after synthesis by the mass of carbon from the total acetlyne input during synthesis. (b) Chemical yield calculated by dividing CNTs total mass collected after synthesis by the total mass of all precursors (acetylene, toluene, ferrocene) input during synthesis. Results are displayed for 15 vol% (blue circle), 5 vol% (green diamond, and 1.5 vol% acetylene (yellow triangle). CNTs total mass collected are normalized for a 10 substrates synthesis; Figure S3. SEM micrographs of VACNT samples obtained on LG-Al and HG-Al substrates from 5 vol% of C_2_H_2_ and for different synthesis durations; Figure S4. External and internal diameters distibutions of CNTs synthesized for (a) 1.5 vol% acetylene (yellow bars) and (b) 15 vol% acetylene (blue bars), for different synthesis duration. External diameters colors are lighter, and internal diameters colors are darker. Distributions are based on around hundred individual CNT for external diameters and fifty for internal diameters; Figure S5. Number of walls in CNT versus external diameters, for (a–c) 15 vol% and (d–f) 1.5 vol% acetylene. Synthesis duration are (a,d) 20 min and (b,e) 80 min and (c,f) 160 min; Figure S6. Typical RAMAN spectrum for VACNT carpet grown for low acetylene content and 20 min synthesis duration. Experimental spectrum is represented by a red dotted line. Specific bands obtained by Voigt deconvolution, represented by plain colored lines. Most important bands are indicated by a black arrow. ID/IG ratio average is found to be 1.6; Figure S7. Carpet density calculated from the sample weights on the exposed face of the LG Al substrates (P5) after synthesis from various acetylene contents performed in the (20–160) min duration range: (a) areal density, (b) volume density, both drawn versus synthesis duration. VACNT on faces in contact with the quartz holder are scratched off, thus enabling to differentiate mass and density contribution between the exposed and the hidden face. When indicated, variation range around the mean value is calculated from at least 2 synthesis experiments performed in the same conditions, and corresponds to the minimum and maximum of the mean value; Figure S8. (a) Carbon conversion yield from acetylene calculated by dividing CNTs total mass collected after synthesis by the mass of carbon from the total acetlyne input during synthesis. (b) Chemical yield calculated by dividing CNTs total mass collected after synthesis by the total mass of all precursors (acetylene, toluene, ferrocene) input during synthesis. CNTs total mass collected are normalized for a 10 substrates synthesis; Figure S9. SEM micrographs of VACNT samples obtained on (a) LG-Al and (b) HG-Al substrates from 1.5 vol% of C_2_H_2_ and for different Fe/C ratio. (c) High magnification of VACNT from carpet grown on HG-Al; Figure S10. External and internal diameters distibutions of CNTs synthesized for different Fe/C ratio. External diameters colors are lighter, and internal diameters colors are darker. Distributions are based on around hundred individual CNT for external diameters and fifty for internal diameters; Figure S11. Number of walls in CNT versus external diameters, for different Fe/C ratio.
