# Supplementary material for: Critical Role of the Acetylene Content and Fe/C Ratio on the Thickness and Density of Vertically Aligned Carbon Nanotubes Grown at Low Temperature by a One-Step Catalytic Chemical Vapor Deposition Process
Source: Nanomaterials (Basel). 2022 Jul 7;12(14):2338. doi: 10.3390/nano12142338 (PMC9316033; doi:10.3390/nano12142338)
Supplement: Supplementary file 1 [file nanomaterials-12-02338-s001.zip › nanomaterials-1776719-supplementary.pdf]

nanomaterials

Supplementary informations

Critical Role of the Acetylene Content and Fe/C Ratio on the Thickness and Density of Vertically Aligned Carbon Nanotubes Grown at Low Temperature by a One-Step Catalytic Chemical Vapor Deposition Process

Antoine Combrisson, Emeline Charon *, Mathieu Pinault, Cécile Reynaud and Martine Mayne-L'Hermite

Nanoscience et Innovation pour les Matériaux, la Biomédecine et l'Energie, Commissariat à l'Energie Atomique, Centre National de la Recherche Scientifique,, Université Paris-Saclay, 91191 Gif-sur-Yvette, France; antoine.combrisson@cea.fr (A.C.); mathieu.pinault@cea.fr (M.P.); cecile.reynaud@cea.fr (C.R.); martine.mayne@cea.fr (M.M.-L.)

* Correspondence: emeline.charon@cea.fr

Citation: Combrisson, A.; Charon, E.; Pinault, M.; Reynaud, C.; Mayne-L'Hermite, M. Critical Role of the Acetylene Content and Fe/C Ratio on the Thickness and Density of Vertically Aligned Carbon Nanotubes Grown at Low Temperature by a One-Step Catalytic Chemical Vapor Deposition Process. *Nanomaterials* **2022**, *12*, 2338. https://doi.org/10.3390/nano12142338

Academic Editor: Ana M. Benito

Received: 1 June 2022
Accepted: 28 June 2022
Published: 7 July 2022

Publisher's Note: MDPI stays neutral with regard to jurisdictional claims in published maps and institutional affiliations.

Copyright: © 2022 by the authors. Submitted for possible open access publication under the terms and conditions of the Creative Commons Attribution (CC BY) license (https://creativecommons.org/licenses/by/4.0/).

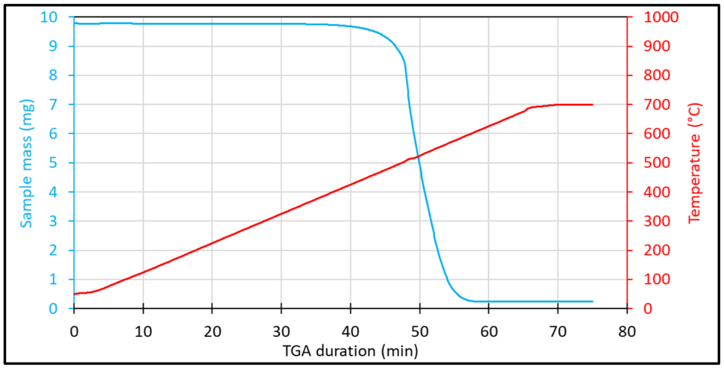

Figure S1. Typical thermo-gravimetric analysis under air indicating the oxidation of VACNT. Temperature, represented by the red curve, rises from room temperature to 700 °C at 10 °C/min. Sample mass during TGA is represented by the blue curve.

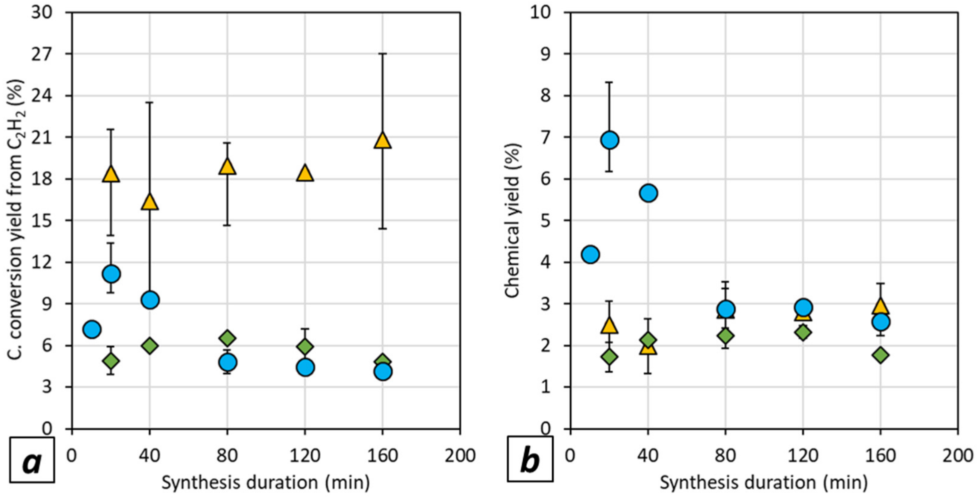

Figure S2. (**a**) Carbon conversion yield from acetylene calculated by dividing CNTs total mass collected after synthesis by the mass of carbon from the total acetlyne input during synthesis. (**b**) Chemical yield calculated by dividing CNTs total mass collected after synthesis by the total mass of all precursors (acetylene, toluene, ferrocene) input during synthesis. Results are displayed for 15 vol % (blue circle), 5 vol % (green diamond, and 1.5 vol % acetylene (yellow triangle). CNTs total mass collected are normalized for a 10 substrates synthesis.

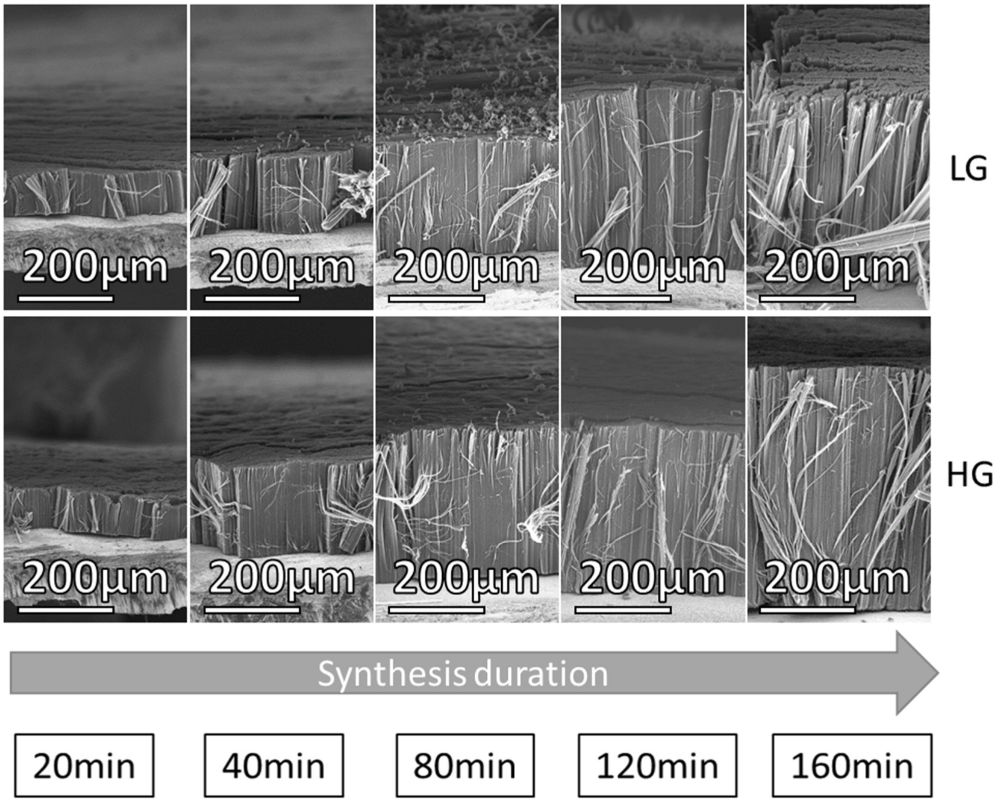

Figure S3. SEM micrographs of VACNT samples obtained on LG-Al and HG-Al substrates from 5 vol % of C_2H_2 and for different synthesis durations.

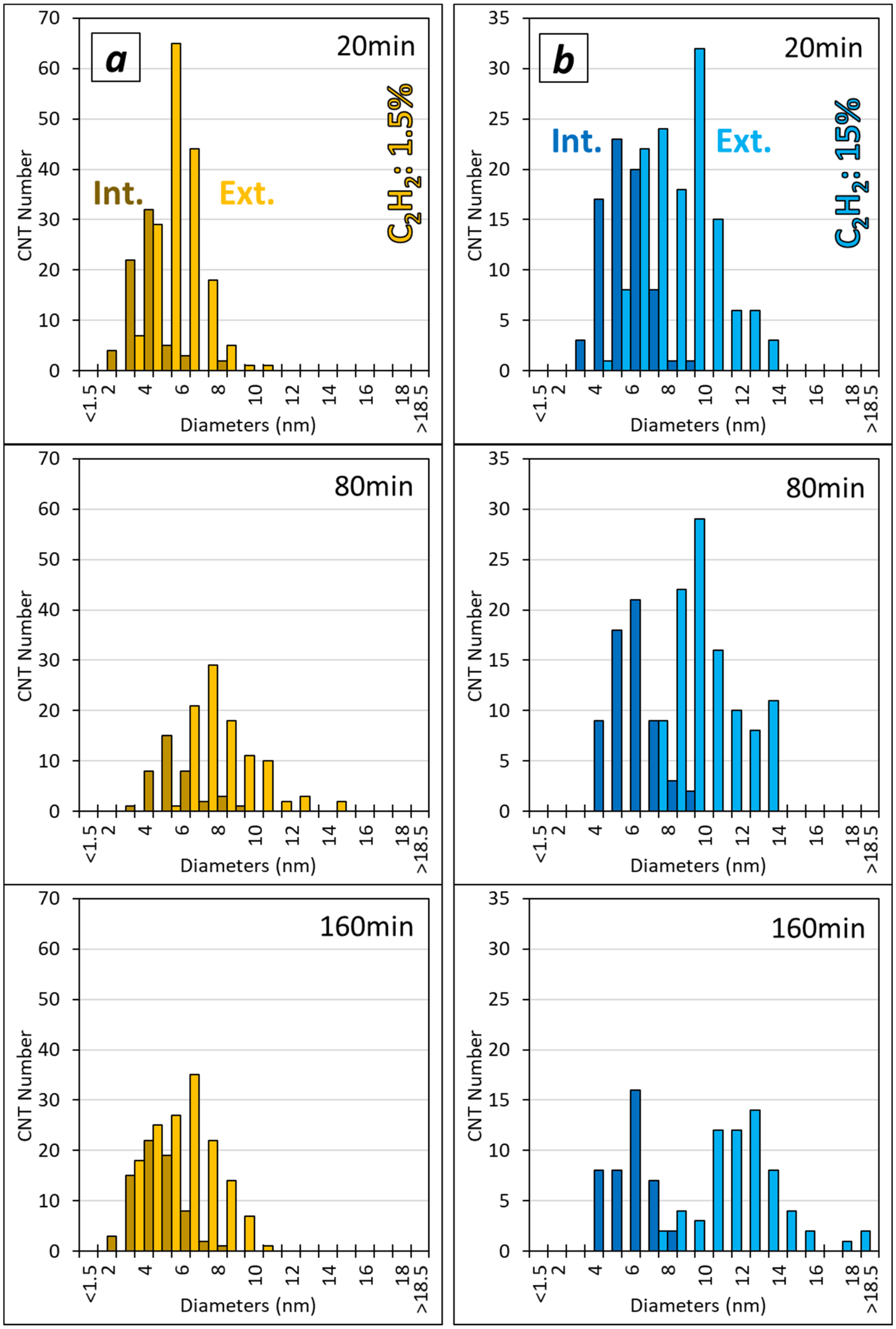

Figure S4. External and internal diameters distibutions of CNTs synthesized for (**a**) 1.5 vol% acetylene (yellow bars) and (**b**) 15 vol% acetylene (blue bars), for different synthesis duration. External diameters colors are lighter, and internal diameters colors are darker. Distributions are based on around hundred individual CNT for external diameters and fifty for internal diameters.

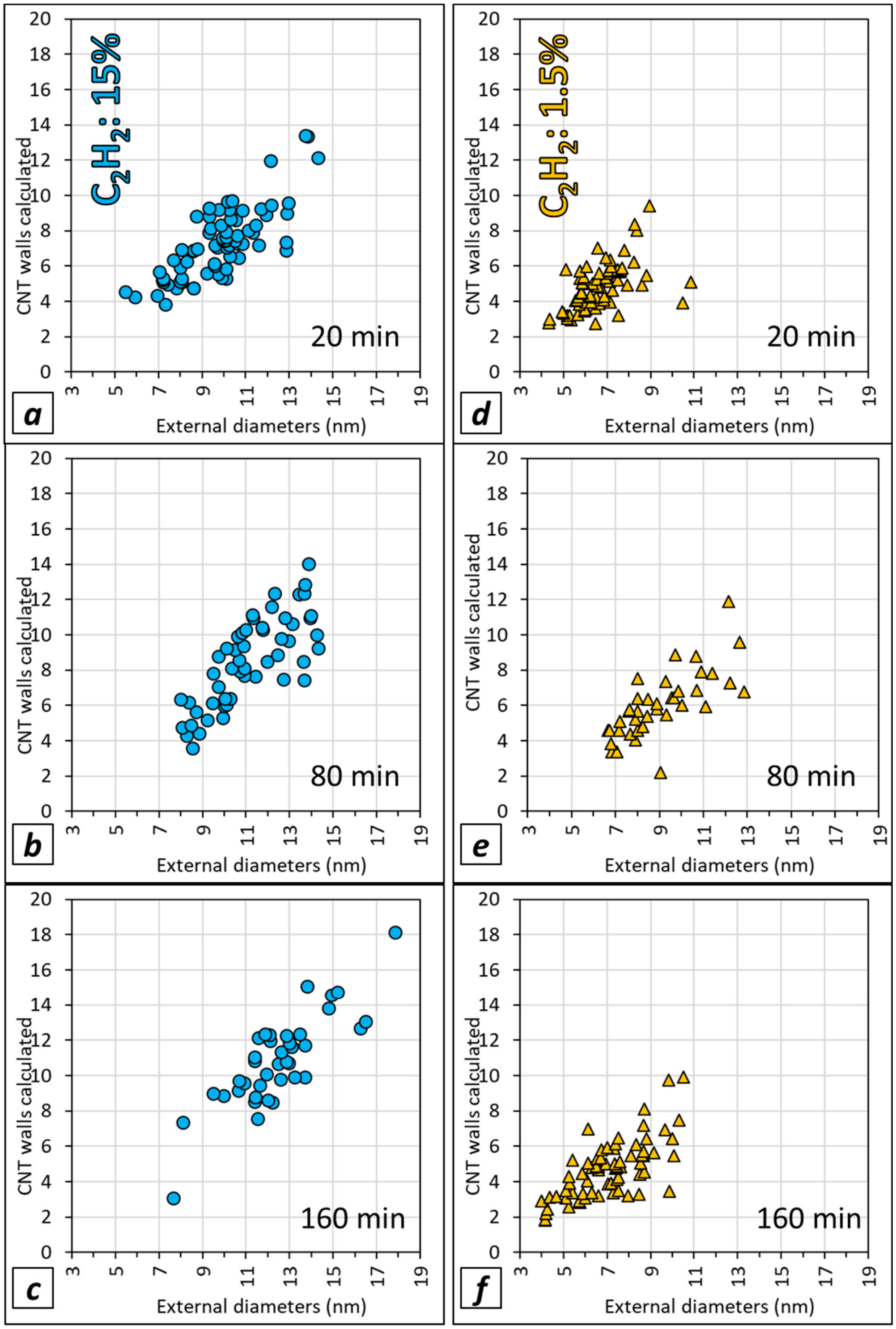

Figure S5. Number of walls in CNT versus external diameters, for (**a**,**b**,**c**) 15 vol% and (**d**,**e**,**f**) 1.5 vol% acetylene. Synthesis duration are (**a**,**d**) 20 minutes and (**b**,**e**) 80 minutes and (**c**,**f**) 160 minutes.

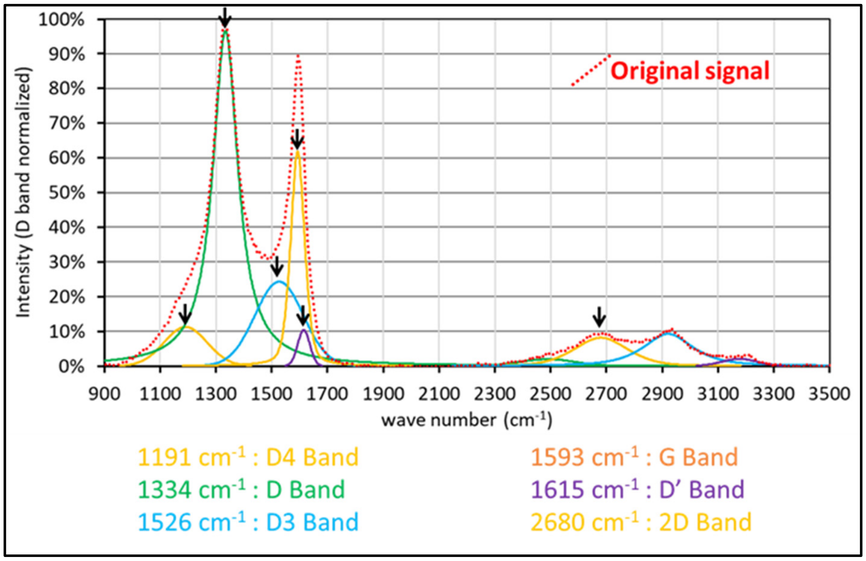

Figure S6. Typical RAMAN spectrum for VACNT carpet grown for low acetylene content and 20 minutes synthesis duration. Experimental spectrum is represented by a red dotted line. Specific bands obtained by Voigt deconvolution, represented by plain colored lines. Most important bands are indicated by a black arrow. ID/IG ratio average is found to be 1.6.

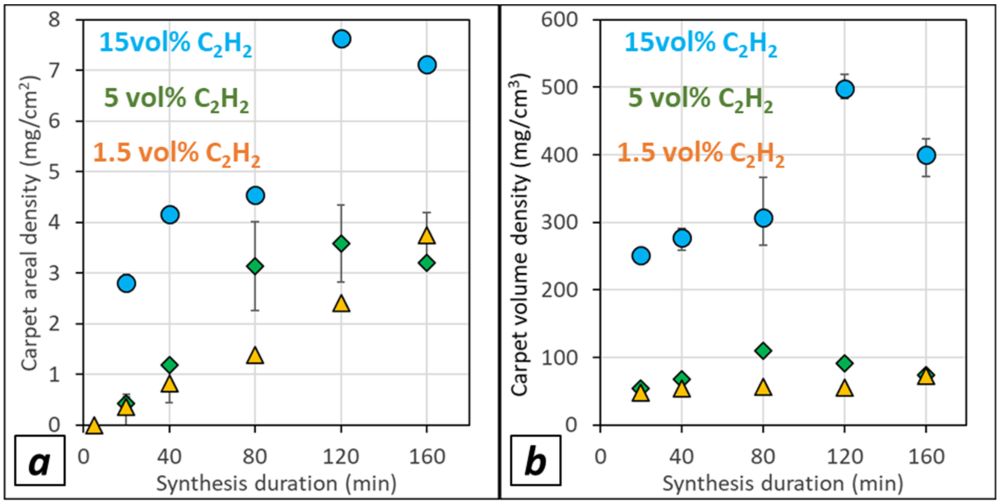

Figure S7. Carpet density calculated from the sample weights on the exposed face of the LG Al substrates (P5) after synthesis from various acetylene contents performed in the (20-160) min duration range: (**a**) areal density, (**b**) volume density, both drawn versus synthesis duration. VACNT on faces in contact with the quartz holder are scratched off, thus enabling to differentiate mass and density contribution between the exposed and the hidden face. When indicated, variation range around the mean value is calculated from at least 2 synthesis experiments performed in the same conditions, and corresponds to the minimum and maximum of the mean value.

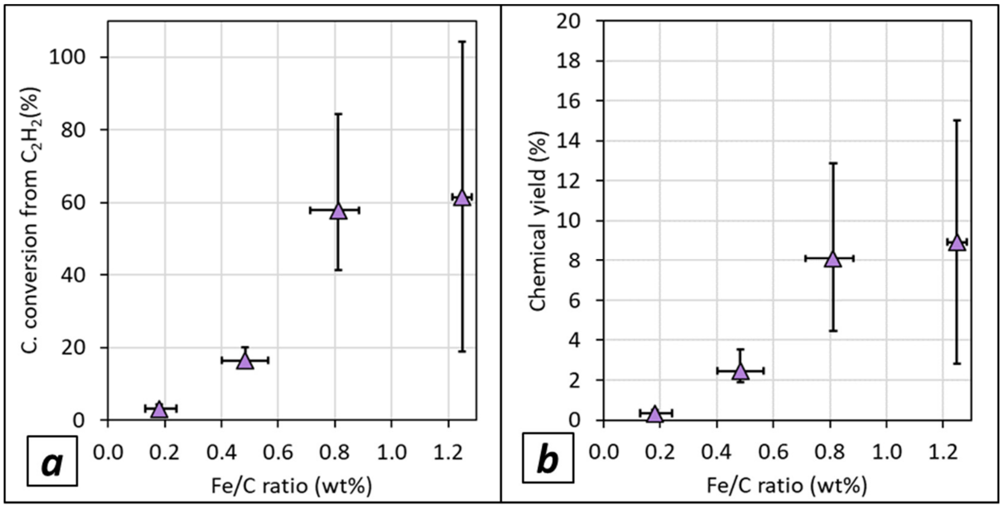

Figure S8. (**a**) Carbon conversion yield from acetylene calculated by dividing CNTs total mass collected after synthesis by the mass of carbon from the total acetlyne input during synthesis. (**b**) Chemical yield calculated by dividing CNTs total mass collected after synthesis by the total mass of all precursors (acetylene, toluene, ferrocene) input during synthesis. CNTs total mass collected are normalized for a 10 substrates synthesis.

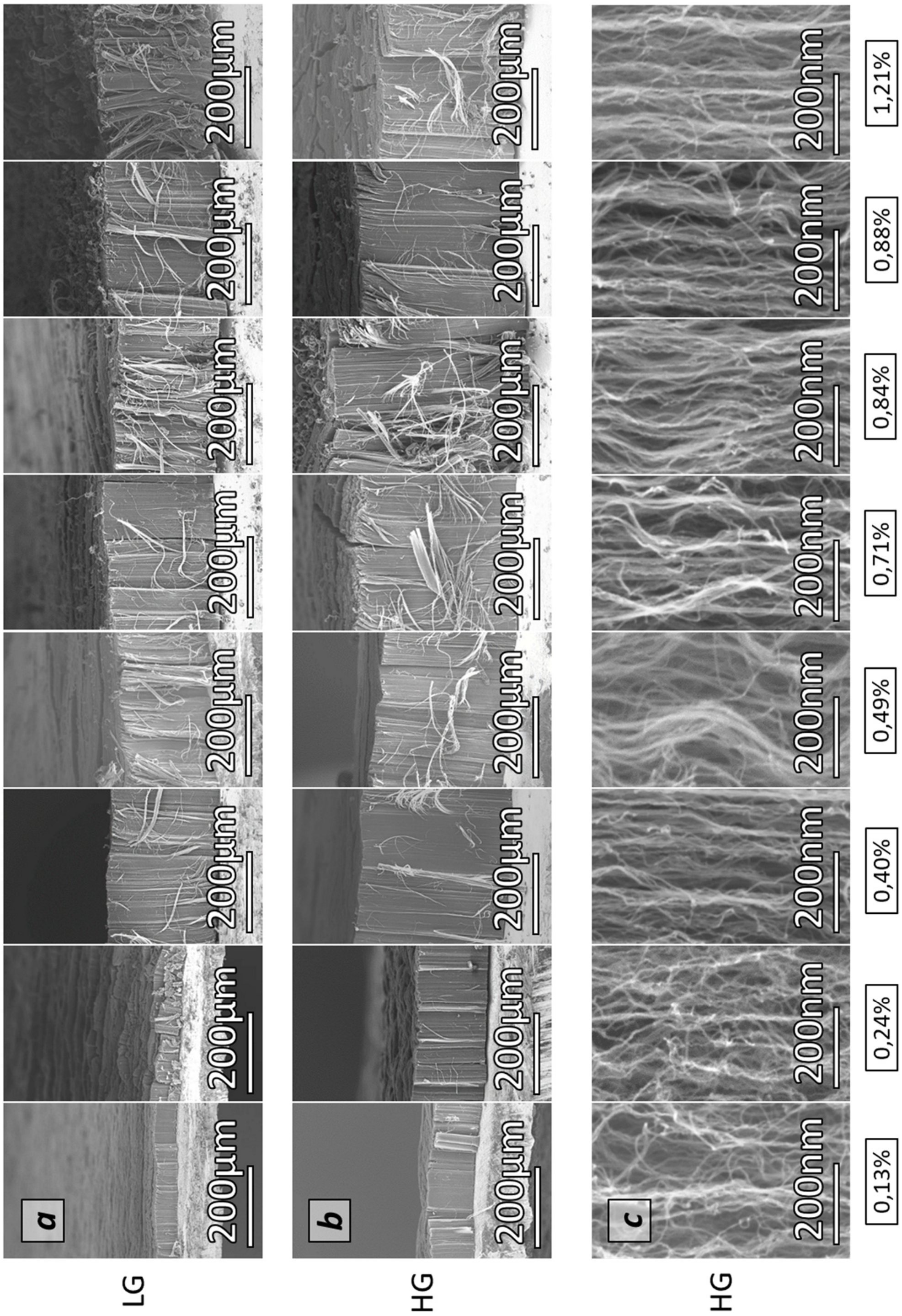

Figure S9. SEM micrographs of VACNT samples obtained on (**a**) LG-Al and (**b**) HG-Al substrates from 1.5 vol % of C_2H_2 and for different Fe/C ratio. (**c**) High magnification of VACNT from carpet grown on HG-Al.

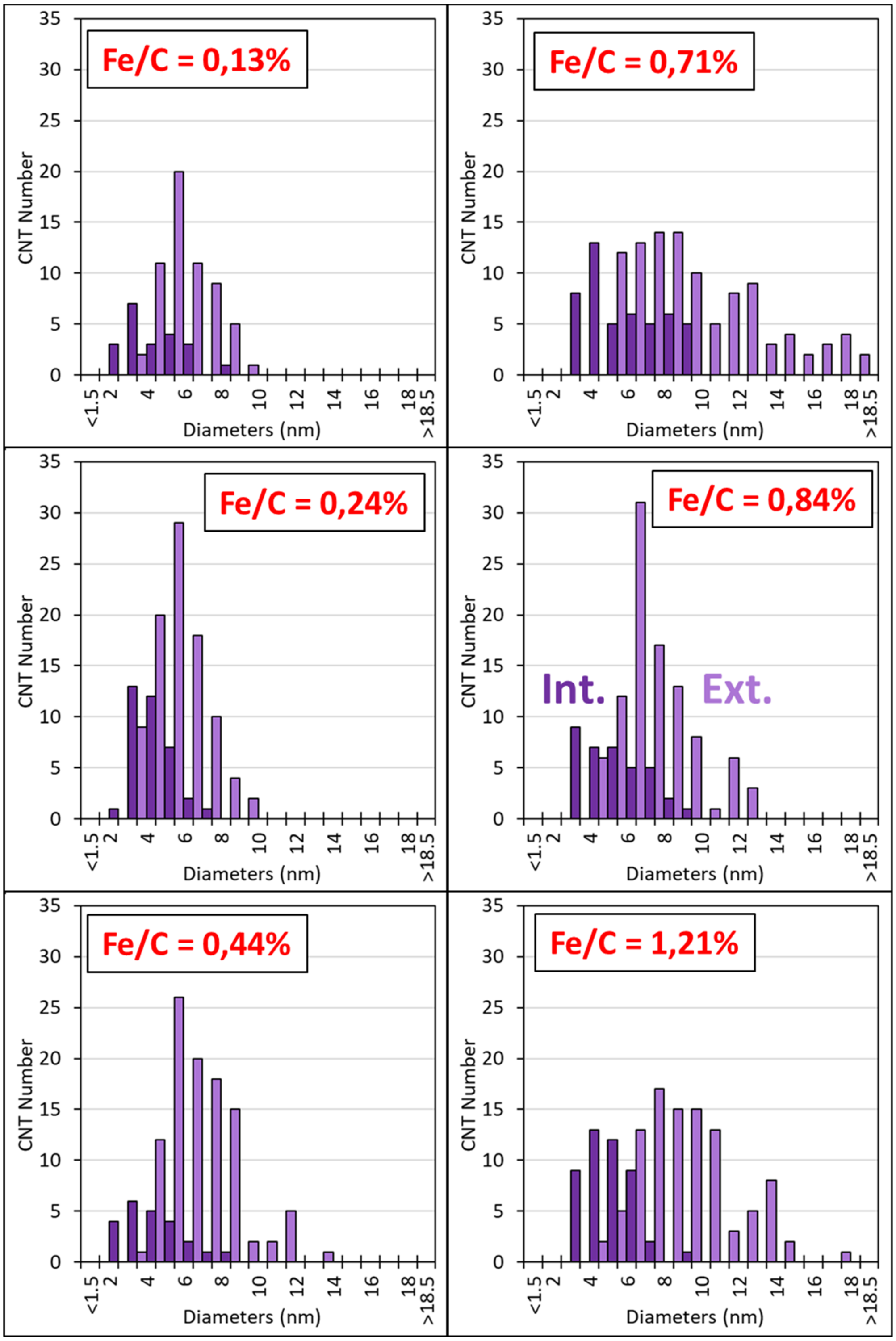

Figure S10. External and internal diameters distibutions of CNTs synthesized for different Fe/C ratio. External diameters colors are lighter, and internal diameters colors are darker. Distributions are based on around hundred individual CNT for external diameters and fifty for internal diameters.

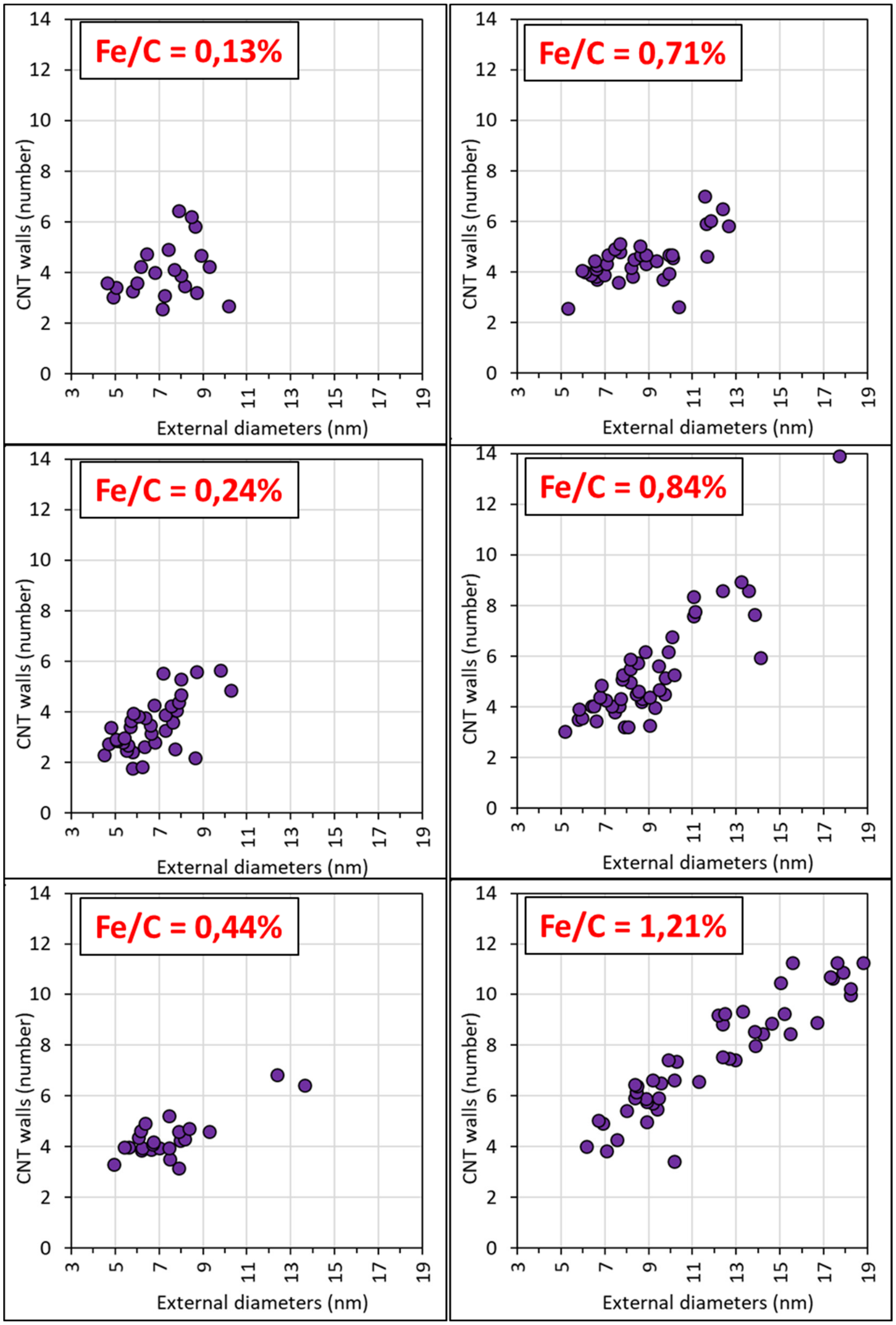

Figure S11. Number of walls in CNT versus external diameters, for different Fe/C ratio.
